# Strength training in soccer with a specific focus on highly trained players

**DOI:** 10.1186/s40798-015-0006-z

**Published:** 2015-04-02

**Authors:** João R Silva, George P Nassis, Antonio Rebelo

**Affiliations:** 1 National Sports Medicine Programme Excellence in Football Project, Aspetar-Qatar Orthopaedic and Sports Medicine Hospital, P.O BOX 29222, Doha, Qatar; 2 Center of Research, Education, Innovation and Intervention in Sport (CIFI2D), Porto, Portugal

## Abstract

**Background:**

Data concerning the physical demands of soccer (e.g., activity pattern) suggest that a high level of performance requires well-developed neuromuscular function (NF). Proficient NF may be relevant to maintain and/or increase players’ short- (intense periods of soccer-specific activity; accelerations, decelerations, and sprinting) and long-term performance during a match and throughout the season.

**Objective:**

This review examines the extent to which distinct modes of strength training improve soccer players’ performance, as well as the effects of concurrent strength and endurance training on the physical capacity of players.

**Data sources:**

A selection of studies was performed in two screening phases. The first phase consisted of identifying articles through a systematic search using relevant databases, including the US National Library of Medicine (PubMed), MEDLINE, and SportDiscus. Several permutations of keywords were utilized (e.g., soccer; strength; power; muscle function), along with the additional scanning of the reference lists of relevant manuscripts. Given the wide range of this review, additional researchers were included. The second phase involved applying six selection criteria to the articles.

**Results and conclusions:**

After the two selection phases, 24 manuscripts involving a total sample of 523 soccer players were considered. Our analysis suggests that professional players need to significantly increase their strength to obtain slight improvements in certain running-based actions (sprint and change of direction speed). Strength training induces greater performance improvements in jump actions than in running-based activities, and these achievements varied according to the motor task [e.g., greater improvements in acceleration (10 m) than in maximal speed (40 m) running movements and in non-squat jump (SJ) than in SSC-based actions (countermovement jump)]. With regard to the strength/power training methods used by soccer players, high-intensity resistance training seems to be more efficient than moderate-intensity resistance training (hypertrophic). From a training frequency perspective, two weekly sessions of strength training are sufficient to increase a player’s force production and muscle power-based actions during pre-season, with one weekly session being adequate to avoid in-season detraining. Nevertheless, to further improve performance during the competitive period, training should incorporate a higher volume of soccer-specific power-based actions that target the neuromuscular system. Combined strength/power training programs involving different movement patterns and an increased focus on soccer-specific power-based actions are preferred over traditional resistance exercises, not only due to their superior efficiency but also due to their ecological value. Strength/power training programs should incorporate a significant number of exercises targeting the efficiency of stretch-shortening-cycle activities and soccer-specific strength-based actions. Manipulation of training surfaces could constitute an important training strategy (e.g., when players are returning from an injury). In addition, given the conditional concurrent nature of the sport, concurrent high-intensity strength and high-intensity endurance training modes (HIT) may enhance a player’s overall performance capacity.

Our analysis suggests that neuromuscular training improves both physiological and physical measures associated with the high-level performance of soccer players.

**Electronic supplementary material:**

The online version of this article (doi:10.1186/s40798-015-0006-z) contains supplementary material, which is available to authorized users.

## Key points

- Neuromuscular training improves both physiological and physical measures associated with high-level performance.
- It seems that strength and power training programs should target all the force-velocity potential/spectrum of the neuromuscular system.
- Due to the conditioned concurrent nature of the sport, combined strength and combined high-intensity training approaches may constitute a good training approach within a football periodized process.

## Review

### Introduction

The central goal of strength/power training in a highly competitive sport is to improve the players’ specific and relevant athletic activities inherent in their sport. To achieve this outcome, different strength/power training modes with i) distinct movement patterns (traditional resistance exercises, ballistic exercises, plyometrics, weight lifting, and/or sport-specific strength-based actions), ii) different combinations of the temporal organization of strength/power training loads (e.g., microcycle and training session variations), iii) distinct loads, iv) a wide range of movement velocities, v) specific biomechanical characteristics, and vi) different training surfaces have been adopted with the final end point of achieving an improvement in players’ performance in relevant motor tasks (e.g., jumping, sprinting, and changing direction) [1-24].

Certain training methods combine different exercise modes (e.g., weight training, plyometric training, and sport-specific force-based actions) and allow for optimal power development and transfer to athletic activities due to both the neural and morphological adaptations typically associated with advanced training [25]. In fact, the intrinsic characteristics of soccer activity patterns (a varied range of motor actions that involve both breaking and propulsive forces as well as distinct contraction modes and velocities that require the all force-velocity potential of the neuromuscular system) that highlight the importance of the principle of specificity in strength and muscle power training cannot be understated [26,27].

A combination of different methods, including high-intensity strength training involving traditional resistance exercises (TRE; squats) and plyometrics [6], TRE and sprint training [10], and complex strength training (CT) [11,15,19], have all recently received considerable attention. Although some similarities exist between the previous modes of strength and power training, there are important differences. In this review, we found that complex training refers to training protocols that are comprised of the alternation of biomechanically comparable strength exercises and sport-specific drills in the same workout (e.g., six repetitions of calf extension exercise at 90% of one repetition maximum (1RM) + 5 s of rest + eight vertical jumps + 5 s of rest + three high ball headers) [25].

By focusing on more effective periodization techniques, researchers have investigated the effectiveness of different loading schemes throughout the power training phase (from high-force/low-velocity end to low-force/high-velocity end or vice versa) [22]. The training-induced effects of exercises with distinct biomechanical and technical characteristics during the plyometric-based component (e.g., purely vertically or a combination of vertically and horizontally oriented exercises [12,16,21,23], as well as the effects of plyometric training on different ground surfaces (grass vs. sand) [12], have both garnered significant attention. Furthermore, the adaptiveness of the functional and muscle structure of professional players (e.g., myosin heavy chain composition) to high-intensity strength training in the isokinetic contraction mode has also been investigated. However, the implementation of this analysis during the off-season resulted in lower ecological validity of these findings [7]. With regard to the search for complementary procedures and/or less stressful interventions, the effects of other methodologies (e.g., effects of electrostimulation training on semi-professional players) on physical fitness have also been investigated [28].

In general, most studies have examined the training-induced performance effects of two [1,6,8,10,14,16,19] to three [2,3,11,12,21,28] sessions per week. Given the multi-component requisites of soccer players’ training (e.g., endurance, speed endurance, strength, power, and agility) that coincide with the increased amount of training time, some researchers examined the short-term effect of a lower weekly volume program (one session) [1,15,19] and the effect of training-induced adaptations of different weekly training frequencies (e.g., one vs. two sessions and one session per week vs. one session every second week) on both physiological and performance parameters during pre-season [19] and throughout the in-season in well-trained soccer players [1].

Nevertheless, despite an increase in the body of evidence regarding the applicability of strength/power training programs to routine soccer training, the short-term duration of interventions (e.g., 4 to 12 weeks) [2,3,6,8,10-12,14-16,19,21-23,28], the wide variety of training methods, the distinct season time lines used throughout the pre-season [2,3,6,12,19] and in-season [8,14-16,21,24,28] periods, the different weekly training loads, and the absence of control groups make the drawing of precise conclusions very difficult. With regard to the latter aspect, it is accepted that due to the importance of winning matches, technical staff of semi-professional and professional teams are unable to implement different training scenarios based on research interests. Nevertheless, in this review, our aim is to contribute to the understanding of the present state of the art of strength/power training and concurrent training in soccer to motivate future studies.

## Methods

### Search strategy: databases and inclusion criteria

The selection of studies was performed in two consecutive screening phases. The first phase consisted of identifying articles through a systematic search using the US National Library of Medicine (PubMed), MEDLINE, and SportDiscus databases. Literature searches were performed from January 2013 until June 2014, and this review comprises papers from 1985 to 2014 (N_1985-2009_ = 76 papers, N_2010_ = 7 papers, N_2011_ = 17 papers, N_2012_ = 4 papers, N_2013_ = 21 papers, N_2014_ = 11 papers). The following keywords were used in combination: ‘elite soccer’, ‘professional soccer’, ‘first division soccer,’ ‘highly trained players,’ ‘seasonal alterations’, ‘performance analysis’, ‘soccer physiology’, ‘football’, ‘strength training’, ‘concurrent training’, ‘training transfer’, ‘neuromuscular performance,’ ‘muscular power’, ‘jump ability’, ‘sprint ability’, ‘agility’, ‘repeated sprint’, ‘intermittent endurance’. Further searching of the relevant literature was performed by using the ‘related citations’ function of PubMed and by scanning the reference lists. The second phase involved applying the selection criteria to the articles. Studies were chosen if they fulfilled the following six selection criteria: (i) the studied athletic population consisted of highly trained soccer players, ii) the players in the sample were not under 17 years of age, (iii) detailed physiological and performance tests were included, iv) the training programs applied were specified, (v) appropriate statistical analyses were used, and (vi) the article was written in the English language and published as an article in a peer-reviewed journal or a peer-review soccer-specific book edition.

### Data extraction and presentation

Data related to the players’ physiological parameters (e.g., lean leg volume, body fat percentage, running economy, anaerobic threshold, maximum absolute and relative oxygen consumption and strength values, peak and mean power values, and rate force development measures) and performance parameters (e.g., soccer-specific endurance tests, maximal aerobic speed, repeated and single sprint tests, jump ability exercises, agility, and ball speed) were extracted. All data are presented as the percentage of change in the means (∆) unless otherwise specified.

### Search data and study characteristics

The aim of providing players with updated data and training approaches in modern scenarios was fulfilled by 23 of the 24 papers published in the last 10 years. There were a total of 24 manuscripts fulfilling the five selection criteria, and the total sample population consisted of 523 soccer players. The distribution of players by competition level was as follows: 322 adults, 145 U-20 players, 12 U-19 players, and 44 U-18 players.

## General physiological considerations of strength/power training

Strength training has become an integral component of the physical preparation for the enhancement of sports performance [29]. While strength is defined as the integrated result of several force-producing muscles performing maximally, either isometrically or dynamically during a single voluntary effort of a defined task, power is the product of force and the inverse of time, i.e., the ability to produce as much force as possible in the shortest possible time [9]. Nevertheless, strength and power are not distinct entities, as power performance is influenced by training methods that maximize both strength and stretch-shortening cycle activity (SSC) [30]. The ability of a muscle to produce force and power is determined by the interaction of biomechanical and physiological factors, such as muscle mechanics (e.g., type of muscle action) and morphological (e.g., muscle fiber type) and neural (e.g., motor unit recruitment) factors, and by the muscle environment itself (e.g., biochemical composition) [31].

The mechanisms underlying strength/power adaptations are largely associated with increases in the cross-sectional area of the muscle (hypertrophy methods) [32]. However, muscular strength increments can be observed without noticeable hypertrophy and serve as the first line of evidence for the neural involvement in the acquisition of muscular strength [32]. Thus, despite the notion that hypertrophy and neural adaptations are the basis of muscle strength development [33], their respective mechanisms of adaptation in the neuromuscular system are distinct [34]. In fact, ‘more strength’, i.e., the adaptational effect, does not necessarily imply an increase in muscle mass, as several distinct adaptations can lead to the same effect [33]. In this regard, the trainable effects of explosive/ballistic and/or heavy-resistance strength training causing enhanced force/power production have been primarily attributed to neural adaptations, such as motor unit recruitment, rate coding (frequency or rate of action potentials), synchronization, and inter-muscular coordination [31,35,36].

### Physiological adaptations in soccer players

**Figure 1:**
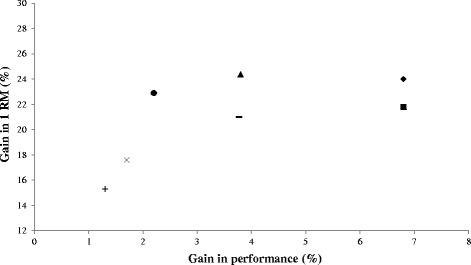
**The gains in strength and d** **ifferent motor abilities of high-level players after 5 to 10 weeks.** Squares represent the average squat jump performance [1,6,14,22]; rhombi represent the average countermovement jump performance [2,22,37]; triangles represent the average four bounce test performance [6]; circles represent the average 10-m sprint performance [2,22,37,38]; x symbols represent the average 40-m sprint performance [1,2,6]; + symbols represent the average change in direction ability [2,38]; and lines represent the average of all the previous motor tasks.

Our analysis suggests that the physiological adaptations underlining strength/power training may result in improvements in different motor tasks and performance qualities in high- and low-level players (Table 1 and Figure 1). In fact, independent of the players’ standard, an enhanced dynamic [1-7,10,14,22,23] and static maximum force production [4,5,28] and increased muscle power outputs during different physical movements can be obtained through the implementation of strength/power training routines [2-8,14,22,37]. Specifically, increases in 1RM were observed during isoinertial assessments of half-squat exercises [1-3,6,10,14,22], hamstring leg curls, and one-leg step-up bench exercises [10]. Additionally, in our analysis, we observed a large range of improvements in the 1RM of well-trained players after short-term intervention periods (e.g., pre-season, Figure. 1, from 11% to 52% during the squat exercise) with average increments of approximately 21% [1-3,6,22,37,38]. Only Helgerud et al. [37] reported considerably larger gains in 1RM compared with other studies (11% to 26%; Table 1). Moreover, increments in maximal isometric voluntary contraction (MIVC) in the leg press task after CT training [11] and in knee extension strength after electrostimulation [28] and isokinetic training [4,5] have also been reported. Interestingly, not only were improvements in absolute force production (1RM) achieved, but an increased efficiency was also evident after allometric scaling of the results; 1RM per lean leg volume (LLV; 1RM/LLV) improved after high- and moderate-intensity modes of strength training [2], and relative force (maximum force divided by body mass) improved after complex strength training [11].

**Table 1:** **Physiological and functional adaptations to strength training**

**Study**	**Level/country/** ***n*** **(age)**	**Type of training**	**F/D**	**P**	**Physiological adaptations**	**Performance changes**
Bogdanis et al., [2]	Professional/Greek/9 (22.9 ± 1.1)	*RST: Program 1 -* 8 to 12 upper and lower body exercises + 4 sets of half-squats at 90% 1RM/5 rep/3-min rest between sets/emphasis on maximal mobilization during concentric action	3×/wk/6 wks	PS	↑17.3% 1RM† ↑16.3% 1RM/LLV† ↑6.2% PPO† ↑5.7% F_0_ (kg) ↔ V_opt_ (ver.min^−1^); V_0_ (ver.min^−1^); LLV	↑ ~1.6% 10m sprint † ↑ ~1.9% 40-m sprint ↑ ~2.5% 10 × 10-m Zig-Zag test (45° COD)† ↑ ~2.1% *t*-test† ↑ ~1% Illinois† ↑ ~10% CMJ†
		*RST: Program 2* - 8 to 12 upper and lower body exercise + 4 sets of half-squat at 70% 1RM/12 rep/1.5-min rest/emphasis on both eccentric and concentric action with controlled movement speed			↑ 4.2% LLV ↑ 11% 1RM ↑ 6.6% 1RM/LLV ↑4.1% PPO ↔ V_opt_ (ver.min^−1^); V_0_ (ver.min^−1^); F_0_ (kg)	↑ ~1% 10-m sprint ↑ ~1.9% 40-m sprint ↑ ~1.3% 10 × 10-m Zig-Zag test (45° COD) ↑ ~1.2% *t*-test ↑ ~0.6% Illinois ↑ ~5.3% CMJ
Bogdanis et al., [3]	Professional/Greek/9 (22.9 ± 1.1)	The *Program 1* adopted in the previous study	3×/wk/6 wks	PS	↑ 5.4% total work in RSA ↑ 10.9% RE ↑ 4.9% VO_2_ max ↑ 7% MAS	↑ 29.4% YYIE2 ↑ 10% DTT
		The *Program 2* adopted in the previous study			↑ 4.5% total work in RSA ↑ 6.2% VO_2_ max ↑ 5.8% VO_2_ max ↔ RE	↑ 21.5% YYIE2 ↑ 9.6% DTT
Loturco et al. [22]	Professional/Brazil/16 (19.8 ± 0.72)	*RST*_(*wk1 to wk3*)_: half-squat exercise during first 3 weeks: wk_1_ - 4 sets × 8 rep (50% 1RM); wk_2_ - 4 sets × 8 rep (65% 1RM); wk_3_ - 4 sets × 8 rep (80% 1RM) *Power training*_(*wk4 to wk6*)_: jump squat exercise: wk_4_ - 4 sets × 4 rep (60% 1RM); wk_5_ - 4 sets × 5 rep (45% 1RM); wk_6_ - 4 sets × 6 rep (30% 1RM)	2×/wk/6 wks	PS	↑ 19.8% 1RM ↑ 18.5% MP_60%-1RM-squat_ ↑ 29.1% MPP_45%-1RM-jump squat_	↑ 4.3% 10m sprint ↑ 7.1% SJ ↑ 6.7% CMJ ↔ 30-m sprint
	Professional/Brazil/9 (19.1 ± 0.7)	*RST*_(*wk1 to wk3*)_: half-squat exercise during first 3 weeks: wk_1_ - 4 sets × 8 rep (50% 1RM); wk_2_ - 4 sets × 8 rep (65% 1RM); wk_3_ - 4 sets × 8 rep (80% 1RM) *Power training*_(*wk4 to wk6*)_: jump squat exercise: wk_4_ - 4 sets × 6 rep (30% 1RM); wk_5_ - 5 sets × 5 rep (45% 1RM); wk_6_ - 4 sets × 4 rep (60% 1RM)	2×/wk/6 wks	PS	↑ 22.1% 1RM ↑ 20.4% MP_60%-1RM-squat_ ↑ 31% MPP_45%-1RM-jump squat_	↑ 1.6% 10m sprint ↑ 4.5% SJ ↑ 6.9% CMJ ↔ 30-m sprint
Ronnestad et al., [6]	Professional/Norway/6 (22 ± 2.5)	*RST* (half-squats): wk_1 to 2_ (3 sets × 6RM); wk_3 to 5_ (4 sets × 5RM); wk_6 to 7_ (5 sets × 4RM) emphasizing maximal mobilization in concentric phase and slower eccentric phase (i.e. ~2 s).	2×/wk/7 wks	PS	↑ 26% 1RM ↑ 9.9% PPO_20kg_ ↑ 11.1% PPO_50kg_ ↔ PPO_35kg_	↑3.6% 4BT ↔ CMJ, SJ; 10m sprint; 30-40m sprint; 40m sprint time
	8 (23 ± 2)	*RST* plus *PT* performed in the same session: *ALB* = [wk_1 to 2_ (3 sets × 8 rep); wk_3_ (3 sets × 8 rep); wk_4 to 5_ (3 sets × 10 rep); wk_6 to 7_ (4 sets × 10 rep)]/*DLHJ* = [wk_1 to 7_ (2 sets × 5 rep)]/*SLFH* = [wk_1 to 7_ (2 sets × 5 rep)] maximal intensity, emphasizing fast switch from eccentric to concentric contraction; 1-min rest between sets	2×/wk/7 wks		↑ 23% 1RM ↑ 10% PPO_20kg_ ↑ 8% PPO_35kg_ ↑ 9.5% PPO_50kg_	↑ 4% 4BT ↑ 9.1% SJ ↑ 0.009% 30- to 40-m sprint time ↑ 1.1% 40-m sprint ↔ CMJ; 10-m sprint
Koundourakis et al., [48]	Professional/Greece/1st league 23 (25.5 ± 1.1)	*Team A* (*high-strength training stress*): *PS*: 11 sessions *RST* + 15 sessions. *SST* + 4 sessions *SAQ* during 7 weeks pre-season; *IN*: 1 sessions RST; 2 sessions SST; 2 sessions SAQ; 1 sessions speed and 1 session reaction speed training during each week of in-season training *RST*: circuit strength training, 10 stations, 4 sets, 10 reps in free weights, 4-min rest between sets; 70% to 80% 1RM; 2 core strength exercises + lunge, squats, steps up on bench with external weight, pullover, arm curls, triceps, and bench press	*PS*: 7 wks *IN*: 35 wks	PS + IN	IN_1_: ↑ 5.3% VO_2_ max ↑ 16.6% BF IN_2_: ↑ 26.4% BF ↔ VO_2_ max	IN_1_: ↑ 7.7% SJ ↑ 7.2% CMJ ↑ 2.2% 10-m sprint ↑ 1% 20-m sprint IN_2_: ↑ 3.8% SJ ↑ 4% CMJ ↑ 1.1%10-m sprint ↑ 0.3% 20-m sprint
	Professional/Greece/1st league 22 (24.7 ± 1.0)	*Team B* (*moderate-strength training stress*): *PS*: 6 sessions RST + 9 sessions SST + 4 sessions. SAQ during 7 weeks pre-season *IN*: 1 session RST/wk; 1 session SST; 1 session SAQ; 1 session speed training during each week of in-season training *RST*: 4 sets; 6 reps, 90% 1RM; explosive action high execution speed; leg extension, hamstring curls chest press, calf raise, pullover arm curls and biceps	*PS*: 7 wks *IN*: 35 wks	PS + IN	*IN*_1_: ↑ 3.9% VO_2_ max ↑ 16.7% BF IN_2_: ↔ VO_2_ max; % BF	IN_1_: ↑ 8.1% SJ ↑ 7.7% CMJ ↑ 2.8% 10-m sprint ↑ 1.6% 20-m sprint IN_2_: ↔ SJ; CMJ; 10- and 20-m sprint
	Professional/Greece/2nd league 22 (23.8 ± 0.9)	*Team C* (*low-strength training stress*) *PS*: 4 session RST + 7 session SST + 4 session SAQ performed during 7 weeks pre-season *IN*: 1 session RST or SST; 1 session SAQ; 1 session speed training during each week of in-season training *RST*: 4 sets; 6 reps, 90% 1RM; explosive action high execution speed, (alternating with SST training every second strength training session); leg extension, hamstring curls chest press, calf raise, pullover arm curls and biceps	*PS*: 7 wks *IN*: 35 wks	PS + IN	IN_1_: ↑ 4% VO_2_ max ↑ 8.7% BF IN_2_: ↔ VO_2_ max; % BF	IN_1_: ↑ 5.9% SJ ↑ 4.8% CMJ ↑ 1.7% 10-m sprint ↑ 0.7% 20-m sprint IN_2_: ↔ SJ; CMJ; 10m and 20-m sprint
Ronnestad et al., [1]	Professional/Norway/ 7 (22 ± 2)	*RST* - *PS*: wk_1 to 3_ (1st session - 3 × 10RM + 2nd session - 3 × 6RM); wk_4 to 6_ (1st session - 3 × 8RM + 2nd session - 3 × 5RM); wk_7 to 10_ (1st session - 3 × 6RM + 2nd session - 3 × 4RM); *IN*: wk_11 to 22_ (1 session wk - 3 × 4RM) half-squats emphasizing maximal mobilization in concentric phase and slower eccentric phase	2×/wk/10 wks + 1×/wk/12 wks	PS + IN	PS: ↑ 19% 1RM IN: ↔ 1RM	PS: ↑ 1.8% 40-m sprint ↑ 3.3% SJ ↔ CMJ; IN: ↔ 40-m sprint; SJ; CMJ
	Professional/Norway/7 (26 ± 2)	*RST* - *PS*: wk_1 to 3_ (1st session - 3 × 10RM + 2nd session - 3 × 6RM); wk_4 to 6_ (1st session - 3 × 8RM + 2nd session - 3 × 5RM); wk_7 to 10_ (1st session - 3 × 6RM + 2nd session - 3 × 4RM); *IN*: wk_11 to 22_ (1 session each 2 wk - 3 × 4RM) half-squats emphasizing maximal mobilization in concentric phase and slower eccentric phase	2×/wk/10 wks + 0.5×/wk/12 wks	PS + IN	PS: ↑ 19% 1RM IN: ↓ 10% 1RM	PS: ↑ 1.8% 40-m sprint ↑ 3.3% SJ ↔ CMJ; IN: ↓ 1.1% 40-m sprint ↔ SJ; CMJ
Chelly et al.,[14]	Junior/NS/11 (17.3 ± 0.5)	*RST* - back half-squat 1st - 1 set × 7 rep 70% 1RM 2nd - 1 set × 4 rep 80% 1RM 3rd - 1 set × 3 rep at 85% 1RM 4th - 1 set × 2 rep 90% 1RM	2×/wk/8 wks	IS	↑ 25% 1RM ↑ 7.2% Wpeak ↔ LMV; TMV, MTCSA	↑ 23% Vfirst step ↑ 7.1% Vfirst 5-m ↑ 12% Vmax ↑ 4.7% 5J ↑ 10% SJ ↔ CMJ; MPV
Kotzamanidis et al., [10]	NS/Greece/12 (17.0 ± 1.1)	*RST* plus *SP* 10-min after strength session: 3 exercises [(Back half-squat at 90° (BHS); step up on a bench with one leg (SU); leg curls for hamstrings (LCH)] wk_1 to 4_ = 4 sets × 8RM + 4 × 30-m; wk_5 to 8_ = 4 sets × 6RM + 5 × 30 m; wk_9_ = 4 sets × 3RM + 6 × 30-m; 3-min rest between sets/3-min rest between sprint rep/10-min interval between strength and sprint program	2×/wk/9 wks	ND	↑ 8.6% 1RM of BHS ↑ 17.5% 1RM of SU ↑ 18% 1RM of LCH	↑ 7.8% SJ† ↑ 6.6% CMJ† ↑ 3.5% 30-m sprint† ↔ DJ40cm
	11 (17.1 ± 1.1)	Only perform the previous defined RST program	2×/wk/9 wks		↑ 10% 1RM of BHS ↑ 16.7% 1RM of SU ↑ 16.1% 1RM of LCH	↔ SJ†; CMJ†; DJ40cm; 30-m sprint†
Los Arcos et al., [23]	Professional/Spain/11 (20.3 ± 1.9)	*RST plus vertical-oriented exercises* (*VS*): *RST* (1 to 2 exercises session) - double (70% to 76% PPO) and single leg (30% to 35% PPO) half-squats (2 sets × 5 reps) and calf exercises (50% to 60% PPO; 2 sets × 5 reps); *VS* (1 to 2 exercises session) - double and single leg CMJ to box (1 to 3 sets × 3 to 5 reps); vertical jump with load (5% BM; 3 sets × 4 reps); skipping and vertical jump (3 sets × 3 reps); drop vertical jump single leg (2 to 3 sets × 3 reps)	12 sessions/5 wks + 3 wks	PS + IS	↑ 12.6% PPO (kg) ↑ 8.1% IT (km.h^−1^)	↔ 5- and 15-m sprint; CMJ; CMJ D; CMJ ND
	Professional/Spain/11 (19.6 ± 1.9)	*RST plus vertical and horizontal oriented exercises* (*VHS*): *RST*: same protocol; *VHS* (1 to 2 exercises session) - sled walking (5 sets × 1 reps × 10 m; 50% to 55% BM); hip extension wall drill single and double (2 sets × 5 reps); horizontal jump with load (3 sets × 3 to 4 reps; 5% BM); drop horizontal jump single leg (2 to 3 sets × 3 reps); sled-towing (maximal speed, 7.5%; 10 m); double-triple jump (1 × 5 reps)	12 sessions/5 wks + 3 wks	PS + IS	↑ 12.2% PPO (kg) ↑ 3.4% IAT (km.h^−1^)	↑ 3.3% CMJ†; ↔ 5- and 15-m sprint; CMJ D; CMJ ND
Aagaard et al., [4]	Elite/Denmark/24 (NS)	*High-resistance isokinetic strength training* 4 sets × 8RM	32 sessions/12 wks	OS	↑ 10% to 26% CON IKE_(0. 4.18 and 5.24 rad/s)_ ↑ 9% to 14% CON IKE_50° (0 and 0.52 rad/s)_ ↑ 5% to 29% PPO_↑ 3.14 rad/s_ ↑ 5% to 29% PPO_50°(↑ 3.14 rad/s)_ ↑ 24% to 42% CON IKE_Vpeak(↑ 5.24 rad/s)_ ↑ 18% to 32% PPO_Vpeak (↑ 5.24 rad/s)_ ↑ MIVC _KE (50°)_	↔ BS _without run up_
		*Low-resistance isokinetic strength training* in isokinetic mode (low-intensity high speed contraction group)4 sets × 24RM			↑ 9% CON IKE_(2.09 rad/s)_ ↔ PPO; PPO_50°;_ MIVC_50°knee extension_; CON IKE_at Vpeak_; PPO_Vpeak ↑ 5.24 rad/s_	↔ BS _without run up_
		*Functional strength training* in the form of loaded kicking movements without ball 4 sets × 16RM			↑ 7% to 13% CON IKE _(0.52-2.09-3.14 rad/s)_ ↑ 9-14% CON IKE_50° (0 and 0.52 rad/s)_ ↑ 7% PPO _(4.18 rad/s)_ ↑ 9-12% PPO_50° (0.52-2.09 to 3.14 rad/s)_ ↔ CON IKE _Vpeak_; PPO_Vpeak(↑ 5.24rad/s)_	↔ BS _without run up_
Maio Alves et al., [19]	Elite/Portugal/9 (17.4 ± 0.6)	*CT*: *1st station*: 6 rep of 90° squats at 85% 1RM then 1 set of 5-m high skipping, in a straight line and then 5-m sprint. 2nd station: 6 rep of calf extension at 90% 1RM then 8 vertical jumps and then 3 high ball headers. 3rd station: 6 rep of leg extension exercise at 80% 1RM then 6 jump from the seated position than 3 drop jumps (60 cm), executing a soccer heading.				↑ 9.2% 5m sprint ↑ 6.2% 15m sprint ↑12.6% SJ ↔ CMJ; 505 agility tests
	8 (17.4 ± 0.6)	The same *CT* training but performed 2× a week				↑ 9.2% 5m sprint ↑ 6.2% 15m sprint ↑12.6% SJ) ↔ CMJ; 505 agility tests
Mujika et al., [15]	Elite/Spain/10 (18 ± 0.5)	*CT*: *1st session* - introduction session of hill sprinting (8% slope); 2nd session - dedicated to sled pulling sprint training, towing ~18% BM; 3rd, 4th, and 5th session (weeks 3 to 5) 3 series of 4 reps of calf rises (~35% BM) and parallel squats (~50% BM) and 2 repetitions per leg of hip flexions (~15% BM); 6th session - stair climbing: 18× (18 steps × 22.5 cm)/120-s rec (alternating single leg, double leg, single, double, frontal, and lateral step). Weight training emphasizing maximal concentric mobilization. Strength and power exercises in sessions 3 to 5 immediately followed soccer-specific activities such as jumps, accelerations, ball kicks, and offensive and defensive actions				↑~2.8% 15m sprint† ↔ CMJ; CMJWAS; CMJ15-S; Agility 15m;
	10 (18 ± 0.7)	*Sprint training*: 1st and 2nd session - 2× (4× 30-m); 3rd and 4th session- 3× (4× 30-m); 5th and 6th session- 4× (4× 30-m); 90-s rec between rep/180-s rec between sets				↔ CMJ; CMJWAS; CMJ15-S; Agility 15m; 15m sprint
Manopoulos et al., [11]	Amateurs/NS/10 (19.9 ± 0.4)	*CT*: *wk*_*1-2*_ : general strength (10 exercises/3 sets/15 to 20 rep); *wk*_*3-4*_ : 3 sets/6 rep (5 different exercises as skipping, jumping on one leg and on both legs, jumping running forwards, backwards and to the side, jumping obstacles and kicking); *wk*_*5-10*_ : (a) 3 sets × 6 instep kicks within a time of 5 s (b) 6 kicking’s with a 5-m run-up approach against resistance provided by a rubber band (RRB) attached on the ankle of the swinging leg (c) 3 × 10-min/5- or 8-a SSG, with or without loads (d) series of modified exercise sequences: *1st*) 6 kicking’s (RRB), 3 jumps, isometry trunk with a player on the back (PB) in a semi-seated position for 6 s, 4 sideward jumps; *2nd*) 6 leg extensions RRB, 3 headers, isometry ankle musculature, carrying PB for 6 s, 1 kicking; *3rd*) 6 knee flexion repetitions RRB, 4 sideward jumps, 3 × 5-m sprints and a soccer kick	3×/wk/10 wks	NS	↑ 13.9% MIVC_leg_ _press_ ↑ 14% MIVC/BW ↑ 29.1% F_60_ ↑ 17.2% F_100_ ↑ 30% EMG VL	↑ ~4% 10-m sprint ↑ ~10% BS _with run up_ ↔ MCS
Impellizzeri et al., [12]	Amateurs/Italian/37 (25 ± 4)	*PT* on grass; *vertical jumping*: 15 sets in wk_1_; 20 sets wk_2_; 25 sets in wk_3 to 4_; always 10 rep per wk; *bounding*: 3 sets wk_1_; 4 sets wk_2_; 5 sets per wk in wk_3-4_; always 10 rep per wk; *broad jumping*: 5 sets × 8 rep wk_1_; 5 sets wk_2_; 7 sets wk_3_; 7 sets wk_4_; always 10 rep per wk_2-4_; *drop jump*: 3 sets × 5 rep wk_1_; 5 sets × 9 rep wk_2_; 6 sets × 15 rep per wk in wk_3-4_; rec 15 to 30 s between repetitions 1 to 2 min between sets	3×/wk/4 wks	PS		↑ 3.7% 10-m sprint ↑ 2.8% 20-m sprint ↑ 4.7% SJ ↑ 14.5% CMJ† ↑ 9% CMJ/SJ†
		Same *PT* protocol but performed on a different ground surface (sand)				↑ 4.3% 10-m sprint ↑ 2.5% 20-m sprint ↑ 10% SJ† ↑ 6.4% CMJ; ↑ 3.7% CMJ/SJ
Sedano et al., [21]	Elite U-19/Spain/11 (18.4 ± 1.1)	*PT*: jump over hurdles: 16 to 26 sets/5 rep; horizontal jumps: 16 to 26 sets/5 rep; lateral jumps over hurdles: 16 to 26 sets/5 rep; wk_1_ - 270 jumps; wk_2,4,9_ - 300 jumps; wk_3,8_ - 240 jumps; wk_5,7_ - 330 jumps; wk_6_ - 180 jumps; wk_10_ - 390 jumps; 30-s rec between sets of 5 rep and 5 min after 4 sets of 5 reps	3×/wk/10 wks	IS		↑ 8% CMJ ↑ 5% CMJWAS ↑ 5.8% BSdl ↑ 6.4% BSndl ↑ 0.32% 10-m sprint ↔ SJ;
Thomas et al., [16]	Semi-professional/UK/12 (17 ± 0.4)	*PT*: DJ_40group_ session began at 80 foot contacts and progressed to 120 by end of training program	2×/wk/6 wks	IS		↑ ~5% CMJ ↑ ~5% 505 agility test ↔ 5-, 10-, 15-, and 20-m sprint time
		*PT*: CMJ_group_ session began at 80 foot contacts and progressed to 120 by end of training program				↑ ~7% CMJ ↑ ~10% 505 agility test ↔ 5-, 10-, 15-, and 20-m sprint time
Gorostiaga et al., [8]	Amateurs/Spain/10 (17.3 ± 0.5)	*Explosive-strength training* (low load weight training and plyometric and sprint exercises): full squat-lift (2 to 3 sets/2 to 6 rep/ 20 to 52 kg) and power clean (3 to 4 sets/ 3 to 4 rep/16 to 28 kg) 2×/wk; vertical CMJ to box (3 to 5 sets/5 to 8 rep/only in wk_1 to 8_); hurdle vertical jumps (3 sets/4 rep/only in the wk_9 to 11_); sprints (1 set/3 to 5 rep/15 to 40 m) performed 1×/wk; 2-min rec between sets and exercises	2×/wk/11 wks	IS	↔ Hr_13-14 km.h−1(bpm)_; ↔ La_13-14 km.h−1(mM)_	↑ 5.1% CMJ ↑ 7.5% CMJ_20kg_ ↑ 13.9% CMJ_30kg_ ↔ 5- and 15-m sprint; CMJ_40-50-60-70kg_
Billot et al., [28]	Amateurs/French/10 (20 ± 2)	*ES*: 2-min session on both quadriceps femoris muscle (36 contractions per session); knee fixed at 60° (0° corresponding to full extension of the leg); EMS 3 s long followed by a rest period of 17 s (duty cycle 15%); intensity range 60 to 120 mA (higher than 60% of muscle voluntary contraction)	3×/wk/5 wks	IS	↑ 22.1% ECC IKE _(−60°.s−1)_ ↑ 9.9% CON IKE _(60°.s−1)_ ↑ 23.2% CON IKE _(240°.s−1)_ ↑ 27.1% MIVC_KE (60°)_	↑ 9.6% BS _without run up_ ↑ 5.6% BS _with run up_ ↔ SJ; CMJ; CMJWAS; 10-m sprint; V_10 m_

↑, significant improvement; ↓, significant decrement; ↔, no significant alterations; †, significant differences between groups; ~, approximately and data extracted from graphs; NS, not specified; F/D, frequency and duration of training protocols; P, period of the soccer season; rec, recovery; RST, resistance strength training; PT, plyometric training; SP, sprint training; wk, week; PS, performed during preseason; IS, performed during in-season; ND, not defined; rep., repetitions; 1RM, one repetition maximum; 1RM/LLV, maximal strength in half-squat strength per lean leg volume; PPO, peak power output; F_0_, individual theoretical maximal force generated at zero pedal speed; V_opt_, speed were the highest value of power is achieve; V_0_, maximal cycling speed corresponding to zero load; LLV, lean leg volume; m, meters; COD, change of direction; CMJ(_10-20-30-40-50-60-70kg)_, countermovement jump with or without external (load); RSA, repeated sprint ability test; RE, running economy; VO_2_ max, maximal oxygen consumption; MAS, maximal aerobic speed; YYIE2, Yo-Yo intermittent endurance test level 2; DTT, Holff’s dribbling track test; MP_60%-1RM-squat_, mean power; MPP_45%-1RM_, jump squat; mean propulsive power; SJ, squat jump; ALB, alternate leg bound; DLHJ, double leg hurdle jump; SLFH, single leg forward hop; 4BT, four bounce test; SST, soccer-specific strength; SAQ, speed, agility and quickness; BF, body fat; Wpeak, leg cycling peak power; LMV, leg muscle volume; TMV, thigh muscle volume; MTCSA, mean thigh cross-sectional area; V_first step_, velocity during the first step after the start of sprint test; V_first5-m_, average running velocity during the first 5 m of the sprint test; Vmax, maximal running velocity; 5J, five jump test; MPV, maximal pedaling velocity; BHS, back half-squat at 90°; SU, step up on a bench with one leg; LCH, leg curls for hamstrings; DJ40cm, drop jump from 40-cm height; VH, vertical oriented exercises; VHS, vertical and horizontal oriented exercises; BM, body mass; IAT, individual anaerobic threshold; CMJ D, countermovement jump dominant leg; CMJ ND, countermovement jump non-dominant leg; OS, off-season; CON IKE_(0. 4.18 and 5.24 rad/s)_, concentric isokinetic knee extensor peak torque (angular velocity)_;_ CON IKE_50° (0 and 0.52 rad/s)_, concentric isokinetic knee extensor peak torque at 50° knee extension (angular velocity); PPO_↑3.14 rad/s_, peak power at angular velocity higher than 3.14 rad/s; PPO_50°(↑3.14 rad/s)_, peak power at 50° knee extension (angular velocity); CON IKE_Vpeak(↑5.24 rad/s)_, concentric isokinetic knee extensor peak torque exerted at the instance of peak velocity (angular velocities higher than 5.24 rad/s); PPO_Vpeak (↑5.24 rad/s)_, peak power output exerted at the instance of peak velocity (angular velocities higher than 5.24 rad/s); MIVC_50°__knee extension_, maximal isometric voluntary contraction of knee extensors (angle); BS _with or without run up_, ball speed after kicking with or without previous run up; CT, complex strength training; CMJWAS, counter movement jump with arm swing; CMJ_15-s_, counter movement jump during 15-s period; RRB, resistance provided by a rubber band; SSG, small sided game; PB, player on the back; MIVC_leg_ _press_, maximal isometric voluntary contraction in the leg press machine (knee and hip angles of 110° and 90°, respectively; 180° = full extension); MIVC/BW, maximal force divided by body weight; F_60−100_, maximal force value during the first 60 or 100 ms of the contraction; EMG VL, electromyography activity of vastus medialis of the swinging leg (phase 3) normalized relatively to the maximal EMG value during kick; MCS, maximal cycling speed; CMJ/SJ, eccentric utilization ratio; BSdl, ball speed after kicking with dominant leg; BSndl, ball speed after kicking with non-dominant leg; UK, United Kingdom; Hr_13- 14 km.h-1(bpm)_, _-_ heart rate at 13 and 14 km.h^−1^; La_13- 14 km.h-1(mM)_, blood lactate concentration at 13 and 14 km.h^−1^; V_10m_, velocity at 10-m sprint.

According to Harris et al. [27], intervention studies should use a specific isoinertial loading scheme, and test protocols should assess performance over the force-velocity continuum to gain a better understanding of the effect of load on muscular function. Moreover, neuromuscular-related qualities, such as impulse, rate of force development (RFD), and explosive strength, can better predict athletic performance; thus, the development of these approaches should be targeted [27]. The functional performance of soccer players seems to be more significantly associated with variables that are measured within the power-training load range (75% to 125% of body weight [BW] in half-squats) at which peak power (PP) is obtained (60% 1RM = 112% of BW) [39]. The PPs of highly trained soccer players were shown to occur with loads of 45% and 60% 1RM during jump- and half-squat exercises, respectively [22,39]. It is likely that superior improvements in power performance may be achieved by working on these optimal power training load ranges [22,39].

One particular muscle strength/power training adaptation involves an increase in the force-velocity relationships and the mechanical parabolic curves of power vs. velocity after high-intensity training programs, both in isoinertial [14] and isokinetic [4] exercises. Ronnestad et al. [6] and Gorostiaga et al. [8] observed increases in the force-velocity curve after high-intensity TRE and explosive-type strength training among professional and amateurs players, respectively. In the former study, the analysis of the pooled groups revealed increases in all measures of PP [6]. It seems that high-intensity strength training significantly increases performance in professional players at both the high-force end (increases in 1RM and sprint acceleration) and the high-velocity end (improvements in peak sprint velocity and four bounce test; 4BT) but only as long as the subjects perform concurrent plyometric and explosive exercises during their soccer sessions [6]. Furthermore, Los Arcos et al. (2013) recently found that professional players performing 5 weeks of pre-season and 3 weeks of in-season strength/power training increased the load at which PP was achieved during the half-squat exercise [11]. Additionally, 10 weeks of complex strength training, consisting of soccer-specific strength and skill exercises (soccer kick), improved measures of explosive strength and RFD during the isometric leg press in low-level players, with an increase in the electromyography (EMG) activity of certain muscles involved in the task also reported [11].

### Adaptations in sport-specific efforts

The effectiveness of a strength/power program is evaluated by the magnitude of sport-specific improvements. Although the predominant activities during training and matches are performed at low and medium intensities, sprints, jumps, duels, and kicking, which are mainly dependent on the maximum strength and anaerobic power of the neuromuscular system, are essential skills [40]. Power and speed usually support the decisive decision-making situations in professional football, e.g., straight sprinting is the most frequent physical action in goal situations [41]. Furthermore, a high degree of stress is imposed on the neuromuscular system of players to enable them to cope with these essential force-based actions required during training and competition (e.g., accelerations and decelerations) [42,43].

**Figure 2:**
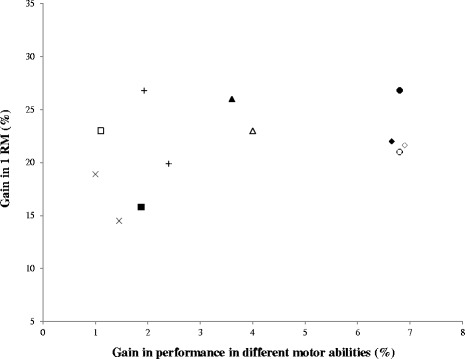
**Gains in strength and motor abilities of high level players after different training modes (5 to 10 weeks).** x and dashed x symbols represent the change of direction ability performance after traditional resistance exercises programs (TRE) [2] and combined programs (COM) [38], respectively; filled and unfilled squares represent the 40-m sprint performance after TRE [1,2] and COM [6], respectively; + and dashed + symbols represent the 10-m sprint performance after TRE [2,37] and COM [22,38], respectively; filled and unfilled triangles represent the four bounce test performance after TRE [6] and COM [6], respectively; filled and unfilled rhombi represent the squat jump performance after TRE [1,14] and COM [6,22], respectively; and filled and unfilled circles represent the countermovement jump performance after TRE [2,37] and COM [22], respectively.

**Figure 3:**
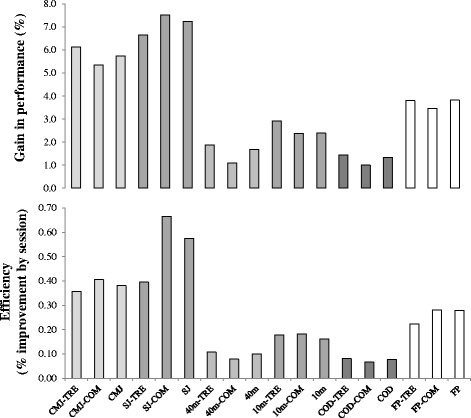
**Percentage of improvement by training program and training session.** Percentage of improvement by training program and training session after traditional resistance exercises programs (TRE), combined programs (COM), and strength/power training programs in the different motor tasks and overall functional performance (FP) of high-level players. Countermovement jump (CMJ) after TRE (CMJ-TRE) [2,20,37]; CMJ after COM (CMJ-COM) [22,23,38]; CMJ [2,20-23,37,38]; squat jump (SJ) after TRE (SJ-TRE) [1,14]; SJ after COM (SJ-COM) [6,19,22]; SJ [1,6,14,19,22]; 40-m sprint performance after TRE (40m-TRE) [1,2]; 40-m sprint performance after COM (40m-COM) [6]; 40-m sprint performance (40-m) [1,2,6]; 10-m sprint performance after TRE (10m-TRE) [2,20,37]; 10-m sprint performance after COM (10m-COM) [22,38]; 10-m sprint performance (10m) [2,20-22,37,38]; change of direction ability (COD) after TRE (COD-TRE) [2]; COD after COM (COD-COM) [38]; COD [2,38]; FP after TRE (FP-TRE) [1,2,6,14,20,37]; FP after COM (FP-COM) [6,19,22,23,38]; and FP [1,2,6,14,19-23,37,38].

Although not universally confirmed, there is evidence of associations between the measures of maximal (1RM) [44] and relative strength (1RM/BM) [45], as well as between certain muscle mechanical properties, such as peak torque [46,47] and PP [39], and the ability of soccer players to perform complex multi-joint dynamic movements, e.g., jumping and sprinting actions. Independently of a player’s level, strength-related interventions represent a powerful training stimulus by promoting adaptations in a wide range of athletic skills (e.g., jumping, Table 1, Figures 1, 2 and 3 and Additional file 1: Figure S1-5) [2,3,6,8,10,12,14,15,19,21-23,48] and soccer-specific skills (soccer kick) [21,28] (Tables 1 and 2). Interestingly, the addition of a long-term strength/power training program to normal soccer training routines seems to result in a higher long-term increase in the physical performance of elite youth players [45,49]. Furthermore, to have a clear picture of the effect of strength training on physical performance, different motor tasks should be assessed; jumping, sprinting, and change of direction abilities may represent separate and independent motor abilities, and concentric and slow SSC jumping actions are shown to be relatively independent of fast SSC abilities [50].

**Table 2:** **Physiological and functional adaptations to concurrent strength and endurance training**

**Study**	**Level/country/** ***n*** **(age)**	**Type of training**	**D**	**P**	**Physiological adaptations**	**Performance changes**
Nunez et al., [13]	Semi-professional/Spain/16 (28 ± 3.7)	*ST* and *ET* - a sequence of general, special, and specific exercises incorporated in different training blocks. ET followed the time-line sequence of variable trajectory, medium extensive, intensive, and short intensive intervals. ST followed the sequence of maximal holds, fast holds, horizontal, and vertical jumps. ET block (2 sessions ET + 1 session ST) ST block (1 session ET + 2 session ST)	4 blocks of 12 wks	S		↑ 73% to 80% Probst test ↑ 11.1% to 16.2% SJ ↑ 8% to 8.7% CMJ ↑ 6% to 7% CMJWAS
Wong et al., [20]	Professional/Hong-Kong/9 (24.6 ± 1.5)	*ST*: 5 exercises; high-pull, jump squat, bench press, back half-squat, and chin-up; 4 sets at 6RM with 3-min rest between sets) *SE*:16 x 15 s at 120% of MAS with 15-s rest	2×/wk/8 wks	PS		↑ 4% VJ, ↑ 5.9% T_10m_ ↑ 2.8% T_30m_ ↑ 19.7% YYIR1, ↑ 3.1% MAS ↑ 9.2% MAS_distance_
Lopez-Segovia et al., [18]	Elite/Spain/ U-19	*ET*: high-intensity runs, physical-technical circuits and SSG, with maximal intensity during 4-6-min periods. *ST*: jumps with and without external training loads, half-squats and full-squats. The speed of movement ranged from 0.8 to 1.2 m.s^−1^. ST complemented with sprint exercises with loads (5 kg) including change of direction movements, and 15- to 20-m take-offs with resisted sled-towing (10 kg)	2×/wk/16 wks	PS-IS		↑ 6.8% CMJ_(20 kg)_ ↑ 5.8% Fsquats_20kg_ ↑ 7.1% Fsquats_30kg_ ↑ 5.2% Fsquats_40kg_ ↓ 2.3% T_20m_ ↓ 2.4% T_30m_ ↓ 3.2% T_st/10-20-m_ ↓ 1.6% T_st/10-30-m_ ↓ 2.6% T_st/20-30-m_
Helgerud et al., [37]	Elite/Spain/23 (25; range 20 to 31)	ET: 4 × 4 min at a treadmill (5.5% inclination) 90% to 95% HRmax separated by 3-min jogging at 50% to 60% HRmax ST: 4 sets × 4RM half-squats 90° with 3-min rest between sets	2×/wk/8 wks	PS	↑ 8.6% VO_2_ max ↑ 3.7% RE_(11km.h)_ ↑ 52% 1RM_HS_ ↑ 49% 1RM/BW	↑ 3.2% T_10m_ ↑ 1.6% T_20m_ ↑ 5.2% CMJ
Jovanovic et al. [24]	Elite junior/Croatia/50 (19)	RST: 2 session a wk targeting the major muscle groups (e.g., legs, back, and chets) with workouts focusing power development (e.g., jump squat, squats, and bench throws) with loads up to 75% to 85% 1RM; SAQ: 3 sessions a wk, work:rest ratio of 3:2; ET: 1 session a wk, 4 × 4 min at 90% to 95% HRmax, 3 min rec 55% to 65% HRmax)	8 wks (1st 8 wks in-season)	IS		↑ 2.1% T_5m_ ↑ 3.7% T_10m_ ↑ 1% CMJ ↑ 0.8% CJS ↔ SJ, maximal CMJ
McGawley and Andersson [38]	Semi-professional and professional players/Sweden/9 (23 ± 4)	ET + ST Tuesday: RSA + speed endurance (e.g., 2× [7× (30 s on/90 s off)] ~95%, 3-min rest: reps 3 and 6 with ball) + 2nd session: RST (e.g., 3 × 5 cleans, 2 × 10 squats, 3 × 10 nordic hamstrings, 2 × 10 core rotations, 3 × 10 barbell rowing; 75%. 60- to 90-s rest); Thursday: (e.g., 2× [8× 45 s on/12 s off) agility/SAQ circuit] ~95% + 1 session functional strength (e.g., 2 × 8 lunges, 2 × 10 hamstrings kicks, 2 × 8 sideway lunges, 2 × 10 standing chest press, 2 × 10 crunches: 75%, 60 to 90s rest); Friday: (e.g., dribble track 4 × 4 min/3 min active rest; 90% to 95%, alternate ball) + 1 session PT (e.g., 3 × 4 Borzov jumps/3 × 10 core rotations; 3 × 6 bounding jumps/3 × 20 ball bounces; 3 × 15 toe bounding/3 × 20 fast shifting lunges)	3×/wk/5 wks	PS	↑ 7.6% fat (%) ↑ 6% fat (kg) ↑ 1.5% lean mass (%) ↑ 3% lean mass (kg) ↑ 18.7% 1RM half-squat ↑ 28.5% 1RM lunge ↑ 97.3% iliopsoas (°) ↑ 5.3% hamstrings (°)	↑ 1.4% T_10m_ ↑ 7% CMJ ↑ 1.1% agility ↑ 1.9% RSA ↑ 19.6% perf dec RSA ↑ 15.4% YYIR2 ↑ 65.3% chins ↑ 14.5% hanging sit-ups
	Semi-professional and professional players/ Sweden/ 9 (23 ± 4)	ST + ET the same daily training but the inverse order (1st, the strength training and after endurance training)	3×/wk/7 wks	PS	↑ 7.1% fat (%) ↑ 5.2% fat (kg) ↑ 1.6% lean (%) ↑ 3.6% lean (kg) ↑ 19.1% 1RM half-squat ↑ 19.1% 1RM lunge ↑ 165.2% iliopsoas (°) ↑ 10.3% hamstrings (°)	↑ 2.2% T_10m_ ↑ 1.9% CMJ ↑ 0.9% agility ↑ 0.8% RSA ↑ 16.8% perf dec RSA ↑ 22.9% YYIR2 ↑ 22.9% chins ↑ 9.7% hanging sit-ups

↑, significant improvement; ↓, significant decrement; ↔, no significant alterations; **~**, approximately; NS**,** not specified; F/D**,** frequency and duration of training protocols; P**,** period of the soccer season; ST, strength training; ET, endurance training; SJ, squat jump; CMJ, countermovement jump; CMJWAS, countermovement jump with arm swing; MAS, maximal aerobic speed; VJ, vertical jump; Fsquats_(20-40kg)_, speed of movement during full squats exercise (range of the external load); T_5-30m_, sprint performance; T_st/10-30_, sprint performance in predetermined split distances; VO_2_ max, maximal oxygen consumption; RE _(11km.h)_, running economy (velocity); 1RM_HS_. one repetition maxim in half-squat strength exercise; 1RM/BW, strength per kilogram of body weight; rec, recovery; CJS, continuous jumps with legs extended; YYIR1, Yo-Yo intermittent recovery level one; MAS_distance_, maximal aerobic distance; SSG, small-sided game; CMJ_(20kg)_, countermovement jump (external load); SAQ**,** speed, agility and quickness; HRmax, maximal heart rate; IS**,** performed during in-season; RSA**,** repeated sprint ability; PT**,** plyometric training; perf dec RSA, performance decrement in the repeated sprint ability test; YYIR2**,** Yo-Yo intermittent recovery level 2.

#### Sprint ability

With regard to adaptations in sprint qualities (e.g., acceleration and maximal speed, Table 1 and Additional file 1: Figure S1), improvements in different sprint distances (5- to 40-m distances) [1,2,6,10-12,14,15,19,21,22,48,51] have been reported in different levels of players. On average, highly trained players [1,2,6,22,37,38] need to increase their 1RM half-squat by 23.5% to achieve an approximately 2% improvement in sprint performance at 10- and 40-m distances (Figure 2). Excluding the study of Helgerud et al. [37], which reported significantly larger increments in strength, studies have demonstrated that lower increments in 1RM (19%) are required to achieve a similar improvement in sprint performance (1.9%) after short-term training interventions (in average, an 18% increments in 1RM resulted in a 2% average improvements in 10-m sprint performance [2,22,38] and 17% average increments in 1RM resulted in 1.6% improvements in 40-m distance time [1,2,6]). Nevertheless, improvements in sprint performance have not been entirely confirmed [1,6,8,10,16,22,28]. Notwithstanding, factors associated with the training status of various players, players’ background, and/or the characteristics of the training modes adopted should be considered as the most likely factors. For example, the sole performance of one type of plyometric exercise [16] and of electrostimulation training [28], which has an apparent lower level of specificity, may explain, at least in part, the lack of transfer of training adaptations to dynamic and complex activities, where the coordination and force production of different body muscles, as is the case of sprint performance, are essential.

#### Jump ability

Our analysis suggests that strength/power training induces adaptations in the jump abilities of high-level players (Table 1 and Figure 1 and Additional file 1: Figure S2). On average, 24.4% 1RM improvements during squats result in a CMJ increase of approximately 6.8% [2,22,37]. Lower performance improvements in four bounce test (4-BT; 3.8%) were found with similar increments in 1RM (24.5%) [6], and similar improvements in SJ (6.8%) occurred with an average 1RM increase of 21.8% [1,6,14,22]. Curiously, the plotted data of all studies assessing the improvement in jump abilities in high-level players revealed that, on average (Figure 2, Additional file 1: Figure S5), a 23.5% 1RM increase may result in a 6.2% improvement in jump ability tasks after 6 to 10 weeks of strength/power training [1,2,6,14,22,37]. The previous results suggest that, on average, higher increments in force are needed to improve CMJ to the same extent as SJ (figure 1). This result may reflect the fact that the current programs were not able to increase (at the same relative rate) performance ability in the positive and negative phases of the SSC component and may explain, at least in part, the smaller improvements in sprint performance.

Improvements in the squat jump (SJ) [1,10,12,14,19,22], four bounce test (4BT) [6], five jump test (5-JT) [14], countermovement jump test (CMJ) [2,8,10,12,16,21,22], CMJ with free arms [21], and eccentric utilization ratio (CMJ/SJ) [12] have been observed in different players. Nevertheless, contradictions regarding improvements in SJ after plyometric [21] and in CMJ after high-intensity strength protocols performed by well-trained players can be found in the literature [1,14]. Additionally, no significant increases in CMJ were observed after CT involving workouts with high [19] or low loads [15] or in drop jumps from a 40-cm height (DJ_40_) [10] following TRE and TRE plus sprint training.

#### Change of direction speed (COD)

According to the literature, it is difficult to discern which force/power qualities (e.g., horizontal and lateral) and technical factors influence event- or sport-specific COD ability [52]. To date, limited research has been conducted on agility/COD adaptations, with even less known about high-level athletes. Despite the limitations initially described see Introduction our results suggest that, on average, an increase of 15% in 1RM results in a 1.3% improvement in COD abilities after 5 to 6 weeks of training (Table 1; Figure 1 and Additional file 1: Figure S3) [2,38]. Bogdanis et al. [2] observed that applying TRE-targeting hypertrophic or neural adaptations was effective in increasing COD (Table 1, Additional file 1: Figure S3). Nevertheless, improvements in COD performance evaluated by the 505 agility test after different plyometric techniques [16] were not found after CT [19]. Additionally, in a study by Mujika et al. [15] where players performed CT, no improvements in COD, evaluated by the agility 15-m test, were observed. The spectrum of possible factors associated with this discrepancy in results is ample and includes the players’ background and initial training status, the different training periods during which the intervention was carried out, the structure of the training intervention, game exposure, and distinct force/power qualities and technical factors that influence event- or sport-specific COD. For example, the study of Maio Alves et al. [19] was implemented during pre-season, and the research of Thomas et al. [16] was carried out during in-season. Consequently, the accumulated effect of COD actions performed during training sessions and games may influence these results [46,53]. Although the players are from the same age groups, the differences in the competitive levels of the players from previous studies should not be ignored. Moreover, the lack of improvements in COD after in-season CT that are reported by Mujika et al. [15] may be related to the fact that only six sessions were performed in a 7-week period. As will be further analyzed (‘Training efficiency’), this fact, among others, may suggest that higher training volumes may be necessary to induce adaptations in COD.

#### Sport-specific skills

One of the most important indicators of a successful soccer kick is the speed of the ball. Studies involving amateur players observed that CT [11] and electrostimulation training [28] increase ball speed with [11,28] and without (Table 1) run up [28]. Nevertheless, these improvements were examined in lower standard players. Moreover, elite U-19 players performing plyometric training increased ball speed with the dominant and non-dominant leg [21]. Other studies involving elite players performing different modes of strength training (isokinetic strength training or functional training) did not report improvements in ball speed [4,5]. Nevertheless, in studies performed during the off-season period, training stimulus consists of the exercise mode of the experimental designs and no other types of soccer routines are undertaken. Thus, the results should be analyzed with caution as the scenarios for training transfer to occur during this period are constricted (off-season); the increases in certain strength parameters were not reflected in positive transference to consecutive gains in ball speed.

### Comparing different training variables in strength/power interventions in soccer

The multi-factorial constructs of soccer performance (technical, tactical, and physical performance) and their associated components bring a higher complexity to the designing of the training process. In fact, professionals involved in the preparation of soccer teams have to reflect on several questions associated with the manipulation of the individual variables that affect each of these relevant constructs and how they can affect each other. With regard to physical performance, several potential questions arise: What are the most beneficial movement patterns and type of training? How many sessions do athletes need to improve and maintain the performance outcome? Does ground surface have an effect on adaptations? We will analyze these and other relevant questions in the following sections.

#### Force production and movement pattern specificity: traditional resistance exercises vs. combined programs

Our analysis suggests that the activity patterns of applied exercises may influence performance outcomes (Figures 2 and 3 and Additional file 1: Figure S4 to S5). Therefore, we compared programs involving mainly traditional resistance exercises (TREs) with programs that combine different activity patterns during the training intervention (COM; programs including TRE and ballistic exercises, plyometrics, weight lifting, body weight exercises, and/or sprint training during training cycles). Despite the fact that some limitations can be ruled out from this type of analysis (e.g., differences in session and weekly training volumes and load, the density of different intrinsic activity patterns, and the 1RM percentage used during the loaded exercises), we believe that it will aid in challenging research designs in this field.

##### Effects on sprint performance

On average, despite TRE resulting in superior strength gains compared with COM, greater performance improvements in the 10-m sprint are observed after COM (TRE = in average, 26.8% increments in 1RM resulted in 1.93% average improvements in 10-m sprint [2,37]; COM = in average, 19.9% increments in 1RM resulted in 2.4% average improvements in 10-m sprint [2,22,38]; Figure 2 and Additional file 1: Figure S5). However, our analysis suggests the opposite with regard to 40-m sprint performance (TRE = in average, 15.8% increments in 1RM resulted in 1.9% average improvements in 40-m time [1,2] COM = in average, 23% increments in 1RM resulted in 1.1% average improvements in 40-m sprint time [6]). Nevertheless, all pooled data suggest that despite the TRE result of greater increases in 1RM (26%) than COM (21%), this may not translate into superior improvements in the sprint performance of high-level players (1.9% TRE vs. 2.1% COM; Additional file 1: Figure S4).

##### Effects on jump ability

By performing the same analysis for jump ability exercises (Figure 2 and Additional file 1: Figure S5), we found that there is a tendency toward greater strength increases after TRE (in average, 26.8% increments in 1RM resulted in 6.8% average improvements in CMJ; in average, 22% increments in 1RM resulted in 6.7% average enhancement in SJ; in average, 25% increments in 1RM resulted in 6% average improvements in 4BT) that are not translated into superior performance gains compared with the results observed following COM (in average, 21% increments of 1RM resulted in 6.8% average improvements in CMJ; in average, 22% increments in 1RM resulted in 6.9% average enhancements in SJ; in average, 22% increments of 1RM resulted in 6.4% average improvements in 4BT). In fact, all pooled data show that greater improvements in jump ability may be obtained with lower strength increases after COM than TRE only (Additional file 1: Figure S5; in average, 21.6% increments in 1RM resulted in 6.4% average improvements in jump ability and a 25% average increments in 1RM resulted in 6% average improvements in jump ability, respectively). This higher efficacy of transfer of strength gains to performance improvements after COM seems to be more evident in SSC jump ability (CMJ). Taking into consideration, among other factors, the described associations between physiological and mechanical characteristics (e.g., post-activation potentiation and peak torque) and CMJ and running-based actions in professional players [44,46,54], this fact may suggest that COM may represent a superior method for improving sport-specific actions compared with TRE alone. Additional studies on this topic are necessary.

##### Effects on COD ability

Given the scarcity of literature assessing the effect of COD training modes and the reported small to moderate associations between strength and power variables with COD performance and different characteristics (e.g., test duration, COD number, and primary application of force throughout the test) of the agility tests commonly used to evaluate COD [52], conclusions should be drawn with caution. In fact, within programs involving only TRE, as will be discussed later in this review (‘Manipulation of loading schemes’), it seems that manipulating different mechano-biological descriptors of strength/power stimuli may influence performance adaptations in COD actions [2]. Nevertheless, our analysis shows that, on average, lower strength increases after TRE [2] produce greater performance improvements in the agility *t*-test than after COM [38] (in average, 14.2% increments in 1RM resulted in 1.7% average improvements in *t*-test and a 19.9% average increment in 1RM resulted in 1% average improvement in *t*-test, respectively; Figure 2).

Two studies are particularly relevant with regard to this topic: TRE vs. TRE plus plyometrics [6] and TRE vs. TRE plus sprint training [10]. In the study of Ronnestad et al. [6], although no significant differences between groups were observed, the group of players who utilized combined approaches broadly improved their performance. Additionally, Kotzamanidis et al. [10] observed that the jump and sprint performance of low-level players only improved in the combined program approach. Thus, it seems that combining heavy and light load training schemes may be an effective method for improving muscular function and may be particularly useful when force application is required in a wide range of functional tasks [27].

##### Training efficiency

To estimate the improvement in the different motor tasks and in overall functional performance, as well as the efficiency (efficiency = percentage of improvement/number of training sessions) of strength/power interventions and the effects of the different types of programs (TRE vs. COM) on specific motor tasks and functional performance, we performed an analysis involving all studies in highly trained players where performance outcomes were reported despite no references to changes in force production (Figure 3). Despite the limitations already highlighted, our analysis suggests that even though TRE slightly increases overall functional performance, the efficiency (gains by session) is lower than in COM modes. These uncertainties make this research topic particularly crucial. In summary, considering the high demands of high-level competition, the increase in different motor tasks (1.3% to 7.2%) and overall functional performance (4%) observed in highly trained players following strength/power training programs makes strength/power programs an essential training component. In general, it seems that strength/power training induces greater improvements in jump abilities than in running-based activities. Moreover, combining resistance- and speed-training or plyometric- and soccer-specific strength programs in the same session seems to be more effective than the resistance-training program alone [6,10,48].

#### Manipulation of loading schemes

Bogdanis et al. [2,3] analyzed the effects of high-repetition/moderate-load (hypertrophy) and low-repetition/high-load (neural adaptations) programs on anthropometric, neuromuscular, and endurance performance. These last studies [2,3] and others [4,5,23] suggest that the manipulation of different mechano-biological descriptors of strength/power stimuli (e.g., load magnitude, number of repetitions) is associated with different physiological and performance adaptations in highly trained soccer players. The hypertrophic mode was associated with increases in lower limb muscle mass, while the neural mode was more effective in improving 1RM/LLV, sprint, and COD performance [2]. In another study, Bogdanis et al. [3] found that even though both groups (hypertrophic group vs. neural group) improved the total work performed during a repeated cycle ergometer sprint test (RST; 10 × 6-s sprint with 24-s passive recovery), the neural mode group had a significantly greater improvement in work capacity during the second half (sprint 6 to 10; 8.9% ± 2.6%) compared with the first half of RST (sprint 1 to 5; 3.2% ± 1.7%). These results suggest that the neural mode confers a higher fatigue resistance during RST [3]. In addition, the mean power output expressed per lean leg volume (MPO/LLV) was better maintained during the last six sprint post-training only in the neural group, and there was no change in MPO/LLV in the hypertrophic group in the RST [3]. These results suggest, at least in part, a better efficacy of neural-based programs in high-level players [2,3] that could be linked to several adaptive mechanisms that are not associated with increases in muscle volume. However, the most likely adaptations are at the neuro-physiological level, i.e., changes in the pattern of motor unit recruitment and increases in rate coding [2,32].

Other researchers observed that physiological and performance outcomes can be independent of the kinetics of the power loading scheme used (from the high-force/low-velocity end to the low-force/high-velocity end and vice versa) because the loading scheme components spanned the optimal power training spectrum [22].

#### Contraction modes

The analysis of the impact of high- vs. low-intensity isokinetic strength vs. functional strength showed that professional players who performed a high-load, low angular velocity program had a higher improvement in maximal isometric and isokinetic strength and in PP at different knee angles and velocities [4,5]. Although the increases in dynamic muscle strength were generally associated with the specific velocities used in the training programs, the high-load/low-velocity group also exhibited improvements in muscle force and power at high knee extension velocities [4,5]. Although several explanations can be offered to clarify the greater adaptations associated with a wide range of velocities observed after the high-load/low-velocity strength training program, the most likely explanation is the occurrence of changes in neural and morphological factors associated with this type of training (e.g., increases in RFD, muscle mass, and/or fiber pennation angle).

#### Training frequency

As previously mentioned, high-level soccer players are usually involved in weekly matches of national leagues and are often involved in international commitments, thus limiting the time available for fitness training. Maio Alves et al. [19] found that different weekly volumes (two vs. one session per week) of complex training performed by high-level junior players resulted in similar improvements in sprint, jump, and COD ability. Ronnestad et al. [1] observed that one high-intensity strength training session per week during the first 12 weeks of the in-season period represented a sufficient training stimulus for maintaining the pre-season (two sessions per week for 10 weeks) gains in strength, jump, and sprint performance of professional players. However, a lower weekly in-season volume (one session every two weeks) only prevented detraining in jump performance [1]. Accordingly, a recent study [48] involving a larger sample of players showed that professional teams subject to distinct weekly strength training stress (all performed one resistance strength session a week) exhibit higher neuromuscular performance in the middle of the season than at the start of the season. Nevertheless, only the team that performed a higher number of sessions targeting the neuromuscular system showed improved neuromuscular performance during the second phase of the season. Despite the distinct individual variables that constituted the weekly resistance training session performed by the teams (e.g., percentage of 1RM, number of repetitions and exercises), differences in strength/power training stress were mainly due to the higher employed volume of both soccer-specific strength and sprint sessions [48]. This result again established the important role of the specificity of the training stimulus. Given the important role of circulating levels of androgens in strength and power performance, it is relevant to mention that only the high neuromuscular training scheme positively affected the circulation and activation (increase in 3a Diol G) of the androgen pool (total testosterone) [48].

However, Mujika et al. [15] observed that a low volume of combined forms of strength/power training is more effective in improving sprint performance (15-m sprint time) than the sole performance of lower volumes of sprint training in elite U-19 players.

#### Manipulation of biomechanical components of plyometric-based exercises

Performance outcomes may also be influenced by the biomechanical nature of the exercises employed in a single or combined program. Los Arcos et al. [23] observed that weight training plus plyometric and functional exercises involving vertically and horizontally oriented movements were more effective in enhancing the CMJ performance of highly trained players than exercises involving purely vertically oriented movements. Nevertheless, both groups improved their PP and showed small, although non-significant, improvements in 5- and 15-m sprint performance [23]. In contrast, Thomas et al. [16] examined that both plyometric training involving drop jumps or CMJs were effective in improving the jump (CMJ) and COD ability (505 agility test) of semi-professional players, regardless of the lack of change in short sprint distances. It is important to highlight that although no between-group differences were reported, the improvements in COD ability were twofold greater in the CMJ group. Nevertheless, given the age group of the players (U-18), it is important to be cautious in extrapolating these findings to professional adult players.

#### Training surface

There is also evidence that the ground surface used during plyometrics (sand vs. grass) may influence adaptations [12]. Impellizzeri et al. [12] observed that performing plyometrics on grass produced greater effects in CMJ and in the eccentric utilization ratio CMJ/SJ than when performed on sand. However, a trend toward higher adaptations was observed in SJ when the training program was performed on sand (Table 1). Additionally, sand was found to induce lower levels of muscle soreness compared with grass [12]. The fatigue development and recovery kinetics during and after a game have been well characterized in recent years. A reduction in the players’ ability to produce force toward the end of the match and in the match recovery period, an increase in some indirect markers of muscle damage, and longer periods of post-match muscle soreness have all been described [55-68]. In light of these findings, it may be expected that sandy surfaces may be a good alternative for the execution of plyometric programs during periods of high-volume, high-intensity, or high-frequency training (e.g., pre-season) and when athletes are recovering from injury and trying to regain physical capacity. In fact, in addition to improving neuromuscular capabilities, sand has been shown to produce lower levels of muscle soreness compared with grass [12]. Accordingly, compared with natural grass or artificial turf, the performance of dynamic powerful actions on sand, despite the known higher energy expenditures and metabolic power values, results in smaller impact shocks and limited stretching of the involved muscles [69].

## Interference between concurrent strength and endurance training

Concurrent training involves the incorporation of both resistance and endurance exercises in a designed, periodized training regime [70]. The current dogma is that muscle adaptations to RE are blunted when combined with endurance [71], resulting in lower strength and power gains than those achieved by resistance exercise alone. When the modes of strength and endurance training focus on the same location of adaptation (e.g., peripheral adaptations), the muscle is required to adapt in distinctly different physiological ways [72]. However, when the modes of strength/power and endurance training are at opposite ends of the biomechanical and neuro-coordinative spectrum, the anatomical and performance adaptations may be reduced, and the accuracy of the intended movement, fluidity, and elegance that characterize excellence may be compromised. In fact, it is the entire spectrum of characteristics (e.g., metabolic and neuro-coordinative) of the upstream stimulus (resistance vs. endurance exercise; RE vs. E) that determines the downstream events necessary for training adaptations to occur. The range of factors that may be associated with the interference phenomenon or the incapability of achieving/maintaining higher levels of strength/power during concurrent strength and endurance training is ample and spans from excessive fatigue or increments in catabolic environments to differences in motor unit recruitment patterns, possible shifts in fiber type, and conflicts with the direction of adaptation pathways required by the muscle [34,70,72,73].

### Molecular events

RE stimulates a cascade of events leading to the induction or inhibition of muscle atrophy [74]. From a molecular standpoint, these adaptations result from the downstream events promoted by the phosphatidylinositol 3-kinase/protein kinase B/mammalian target of rapamycin (PI3-k/Akt/mTOR) pathway [74,75]. However, three kinases [p38 mitogen-activated protein kinase (MAPK), AMP-activated protein kinase (AMPK), and calmodulin-dependent protein kinase] are particularly relevant in the signaling pathways that mediate skeletal muscle adaptations to endurance-based training [75,76].

A few studies highlight the notion that both translation efficiency and protein synthesis may be compromised due to the incompatibility of the two different intracellular signaling networks, i.e., activation of AMPK during endurance exercise impairs muscle growth by inhibiting mTOR [74,75]. Nevertheless, other studies revealed that endurance performed after RE did not compromise the signaling pathways of RE (mTORC1-S6K1) [71] and may amplify the adaptive response of mitochondrial biogenesis [76]. Moreover, the translational capacity for protein synthesis can be reinforced rather than compromised when aerobic exercise precedes RE and molecular events are not compromised; mTOR and P70S6K shown greater phosphorylation in response to concurrent aerobic exercise compared with RE alone [77]. Furthermore, chronic concurrent aerobic exercise and RE may increase aerobic capacity and promote a greater increase in muscle size than RE alone [78]. Nevertheless, taking into account the complexity and the several molecular interactions that constitute the cascade of events associated with resistance and endurance exercise, conclusions should be drawn with caution. Additionally, studies have been performed primarily in healthy adults (physically active college students, moderately trained and recreationally active subjects) and not high-level athletes; although not universally confirmed, athletes with more extensive training backgrounds may have distinct phenotypes [79-81] and genotypes than normally active subjects [82]. Moreover, to the authors’ best knowledge, there is no research concerning how the distinct genotypes that can be found within a high-level group of athletes [82-84] may influence the individual responses to concurrent training.

#### Methodological considerations

Given the divergent physiological nature of strength and endurance training [34], the methodology applied, the volume and frequency of training, and the target goal all play key roles in increasing the degree of compatibility between these two key physical fitness determinants [34,72]. Slow long-duration sustained aerobic conditioning (SLDC) has been shown to be potentially detrimental to the overall performance of athletes involved in power sports and, for example, may have a negative impact on strength and power development [85]. Excessive training volumes may contribute to high metabolic stress, leading to high levels of substrate depletion and catabolic states (e.g., increased cortisol responses) [85]. Furthermore, SLDC may compromise recovery and regeneration, leading to a progression in the overtraining continuum [85]. Moreover, the high levels of oxidative stress (e.g., damaging proteins, lipids, and DNA) that are associated with high-volume training may increase reactive oxygen species (ROS) production to a level that overcomes the positive adaptations that may be triggered by ROS, i.e., there is a range in which ROS may represent an optimal redox state for greater performance, as with force production capacity [86]. Additionally, these previous factors associated with SLDC that limit force production may compromise skill acquisition by reducing the quality of execution (e.g., the technical ability of force application) and, thus, motor learning [85]. It is reasonable to consider that there may be certain mechanisms associated with the combination of training modalities that produce positive improvements and are additive in nature [87].

A low-volume, high-intensity approach, such as sprint interval training, may favor an anabolic environment (e.g., growth hormone, insulin-like growth factor-I, IGF binding protein-3, and testosterone) [88-92], maintain a muscle fiber phenotype associated with strength and power capabilities [93], and increase endurance and neuromuscular-related outcomes [94-96]. In fact, HIT and/or combined forms of HIT seem to promote adaptations in skeletal muscle and improvements in laboratory and field endurance-related parameters that are comparable to the effects of high-volume endurance training [94,97-101] and may improve muscle power-based actions [94,102]. Interestingly, the type of previously observed hormonal responses to HIT (e.g., sprint interval training) [88-92] constitutes one of the paradigms of resistance exercise biology, namely, an increase in cellular signaling pathways as well as satellite cell activation that contributes to an increase in translation and transcription processes associated with protein synthesis [74]. In this regard, supramaximal interval training is shown to be superior to high-intensity interval training for concurrent improvements in endurance, sprint, and repeated sprint performance in physically active individuals [103].

Does the magnitude of neuromuscular involvement during training sessions reduce possible incompatibilities associated with concurrent training? Are the biomechanical and neuro-coordinative demands (e.g., accelerations/decelerations impacting mechanical load and neuromuscular demands) of different training modes with similar physiological responses the same (e.g., 4 × 4-min interval running with 2-min rest vs. 4 × 4-min SSG with 2-min rest vs. 4 × 4-min intermittent situational drill with 2-min rest)? It is possible that, from a biomechanical and neuromuscular standpoint, more specific training methods to develop strength/power and endurance performance with higher biomechanical and neuromuscular demands may improve both adaptations and performance outcomes, as well as reduce the negative effect of this interference from a molecular point of view; human-based studies to date are far from agreement regarding the molecular interference after acute concurrent exercise [70]. In fact, strength/power and HIT are characterized by brief intermittent bouts of intense muscle contractions. Questions related to training transfer should be observed with greater attention when extrapolating the applicability of concurrent training to sport-specific settings. In fact, several factors can influence the transfer of strength training in endurance performance and the impact of endurance workloads on strength and power performances [104].

## Soccer: a concurrent modality

A soccer player’s performance is intimately associated with the efficiency of different energy-related systems [105-107]. During the season, players perform intense programs with multiple goals of increasing strength, power, speed, speed endurance, agility, aerobic fitness, and game skills [108]. In fact, despite the predominant activity patterns of the game being aerobic in nature, the most deterministic factors of match outcome depend on anaerobic mechanisms [41]. It is common sense that the most intense match periods and worst-case match scenarios are associated with periods of high mechanical and metabolic stress. In fact, recently developed techniques of match analysis provide a body of evidence that supports the belief that neuromuscular demands of training and competition are higher than initially suspected (e.g., accelerations/decelerations) [42,43,109] and give further support to the viewpoint that strength/power-related qualities are crucial for high-level performance.

There is a belief that by stressing the neuromuscular system, adaptive mechanisms that are neurological, morphological, and biomechanical in nature will be triggered, thus increasing the player’s neuromuscular performance and providing him/her with a superior short- and long-term endurance capacity [17,110-113]. In this regard, associations between neuromuscular qualities (e.g., CMJ peak power) and intermittent endurance exercise [114] and repeated sprint ability performance [115] have also been observed. Moreover, there has been evidence supporting the association between team success and jump abilities (e.g., CMJ and SJ) [116]. Additionally, starter players demonstrate higher strength [108] and power performance capabilities than non-starters [117], and greater neuromuscular capabilities have been associated with game-related physical parameters and lower fatigue development during matches [118]. Moreover, Meister et al. [119] observed that after a match congestion period, players with a higher exposure time show better scores in certain neuromuscular parameters (CMJ, drop jump height, and drop jump contact) than players with a lower exposure time, although this result is not significant. Interestingly, recent reports revealed that neuromuscular-based actions, such as sprinting, have improved more in recent years than physiological endurance parameters. Professional players tested during the 2006 to 2012 seasons actually had a 3.2% lower VO_2_ max than those tested during 2000 to 2006 [120,121]. Although with the obvious limitations and the universal consensus of the importance of aerobic fitness in soccer, these observations suggest that anaerobic power is ‘*stealing space*’ from aerobic power with regard to the constructs relevant in soccer performance. All of these previous facts highlight the role of neuromuscular exercise during soccer training and suggest that soccer routines should be performed concurrently as they are concurrent by nature. In fact, the physiological systems associated with endurance fitness development and maintenance are generally largely targeted in any match competition, friendly game, tactical exercise, circuit technical drills that often involve frequent displacements, and/or small side game exercises performed during a 90-min soccer competition/training session [106,122,123].

### Physiological and performance adaptations

The summary of changes in physiological and functional parameters resulting from concurrent strength and endurance training are presented in Table 2. Wong et al. [20] observed that 8 weeks of pre-season high-intensity strength training and SE resulted in a significant improvement in endurance markers, soccer-specific endurance (SSE), and soccer-specific neuromuscular (SSN) parameters. Helgerud et al. found that 8 weeks of other modes of HIT (aerobic high-intensity training) and high-intensity strength training during the preseason of non-elite [51] and elite [37] football players improved VO_2_ max (8.6% and 8.9%), running economy (3.5% and 4.7%), and 1RM during half-squat strength exercise (52%), respectively. Moreover, the 10- and 20-m sprint performance (3.2% and 1.6%, respectively) and CMJ (5.2%) of elite players also improved [37]. These strength improvements occurred with minor increases in body mass (average 1%) and a substantial increase in relative strength [37]. More recently, McGawley et al. [38] found that a high-frequency program (three times a week) of concurrent high-intensity running-based training with strength/power-based training in the same session resulted in a positive training effect on all evaluated measures, ranging from flexibility, anthropometric, endurance, and neuromuscular-related parameters (Table 2). Moreover, these results suggested that the order of completion of the program, E + RE or RE + E, did not influence the performance adaptations. These results [38] and others [2,37] may support, at least in part, the better compatibility between high-intensity modes of strength and endurance training.

It is reasonable to assume that the players in the studies examining the effects of strength training programs (Table 1) had performed training with significantly high weekly endurance-based loads (e.g., pre-season). In this regard, Bogdanis et al. [3], when examining the strength training effects of the hypertrophic and neural modes in professional soccer players during pre-season, reported that the weekly cycle also involved a considerable amount of interval training and small-sided games, which have been described as effective methodologies targeting endurance fitness and SSE development (for a review, see [95,122]). The authors [3] observed that both aerobic fitness parameters (e.g., VO_2_ max and MAS) and SSE, evaluated by the Yo-Yo intermittent endurance test and Hoff’s dribbling track test, respectively, were significantly improved in both groups (Table 1). Furthermore, other researchers [23] found that strength/power training performed in parallel with endurance training resulted in improvements in the individual anaerobic threshold and muscle/power parameters. Additionally, the performance of explosive-type strength training with routine soccer training did not interfere with the aerobic capacity of amateur young players [8], e.g., sub-maximal blood lactate values. These findings suggest that performing concurrent strength/power training and routine soccer training is advisable because, in addition to an increase in neuromuscular performance and the anabolic environment, this training did not interfere with the development of aerobic capacity [8]. Nevertheless, the question of whether this compatibility is related to the type of endurance and strength performed is highlighted in the distinct between-group results presented in the study of Bogdanis et al. [3], e.g., point ‘Manipulation of loading schemes’, where only the neural group significantly improved with respect to running economy and a trend toward a better performance in the YYIE2 in the neural group than in the hypertrophic group was reported.

In another study [13], semi-professional male soccer players performed both endurance and strength sessions as part of the annual periodization (four cycles of 12 weeks). This type of periodization was effective in improving both the endurance performance (Probst test) and SSN parameters, e.g., CMJ. These results suggested that no adaptation conflicts occur when one or two sessions of strength/power and endurance are simultaneously combined during a soccer training cycle (endurance block composed of two endurance training sessions and one strength training session and vice versa).

Additionally, Lopez-Segovia et al. [18] examined training adaptations in elite U-19 players during a 4-month period. The training program consisted of four sessions per week, targeting the improvement of player’s aerobic performance. Training was complemented with one or two specific strength training sessions per week performed at the start of the training session. This type of periodization improved loaded CMJ performance and the speed of movement in full squats, with loads ranging from 20 to 40 kg. Nevertheless, significant decrements in different sprint abilities were found. According to the researchers, the lack of improvement in the former sprint variables was attributed to the high volume of aerobic work performed. Nevertheless, an increase in MAS (3.2%) was observed after the intervention period [18].

## Conclusions

Our analysis suggests that, independent of the methodology applied (Table 1) and the form of concurrent endurance and strength/power training (Table 2), pre-season training resulted in an improvement in physiological and soccer-specific and non-specific performance parameters. The large responsiveness to training may be associated with the fact that most of the studies were conducted during an early stage of pre-season, with off-season detraining negatively affecting several physical attributes, such as anthropometric characteristics (e.g., decreases in LBM and increases in BF) [124-126], endurance-related markers [53,101,126,127], soccer-specific endurance [101,128], and neuromuscular parameters [126,129]. With this in mind, the overall conclusion of the analyzed literature is that the addition of strength/power training programs to routine soccer training favors a more integral physical fitness development of the player. The associated improvements in physiological (e.g., 1RM/LLV, PP) and performance (e.g., jump, sprint, COD) parameters may, at least in part, increase a player’s ability to cope with training and competition demands. Our analysis suggests that high-intensity strength training (HIST) may be a more efficient method than moderate-intensity methods (hypertrophic). In addition, the compatibility between strength and endurance training may be greater when high-intensity or explosive strength training is combined with high-intensity endurance training to favor a more soccer-specific phenotype.

One of the most sensitive periods of training implementation is the in-season period. As the match is the most important part of the soccer-training schedule, technical staff often view the in-season periodization with particular prudence. They want to maintain or even increase the pre-season gains obtained throughout the short pre-season period (5 to 7 weeks). However, they face the constant dilemma of determining the proper dose/response that allows for the cycle of training-recovering/competing-recovering to be effective; a high volume of training and/or competition interspersed by insufficient recovery favors fatigue development [130], resulting in a transition from a functional to a non-functional overreaching state or, in more severe cases, an overtraining state [131,132]. Unfortunately, studies implemented during in-season are scarce [1,8,13-16,18,21,24,28,48]; seven were conducted with U-19 players, and only four were conducted with adult soccer players [1,13,28,48]. Our analysis suggests that two weekly sessions allow for highly trained players to obtain significant performance enhancements and that one session a week is sufficient to avoid in-season detraining. It may be possible that, in parallel with a higher volume of neuromuscular training (soccer-specific strength/power-based efforts), further in-season improvements could be observed. Moreover, manipulations of the training surface could constitute an important strategy (e.g., players returning from injury and the management of biochemical and perceptual disturbances).

We found that the results of high-force increments vs. low-performance enhancements and the respective efficiency of the programs (jump vs. running-based actions and non-SSC abilities (SJ) vs. SSC-based actions (e.g., CMJ)) suggest that current approaches may overlook some essential aspects required to achieve an increase in a player’s performance capacities. According to Komi [133], an effective SSC is obtained with ‘a well-timed pre-activation of the muscle(s) before the eccentric phase, a short and fast eccentric phase, and an immediate transition (short delay) between stretch (eccentric) and shortening (concentric phase).’ The observed increments in force production will most likely occur to a greater extent in the positive phase of the SSC. We suggest that to achieve greater improvements, weight training should be combined with more soccer-specific strength exercises (e.g., the player’s ability to use strength and power effectively and consistently [134], allowing for the application of force/power in a larger range of planes (horizontal) and specific angles). Therefore, a conditioning method such as Speed, Agility and Quickness (SAQ) may be useful, as it incorporates plyometric and soccer-specific strength exercises and can, therefore, constitute a good conditioning tool for this type of outcome (acting on the entire spectrum of the SSC and on the transition from eccentric to concentric movements; it should be kept in mind that plyometric training is a technique demonstrated to increase musculo-tendinous stiffness, which can optimize power output in explosive movements) [135]. The greater ecological validity of COM approaches make combined methods a preferred training strategy for strength training in soccer; targeting the intra- and inter-muscular aspects of athletic performance should occur in parallel and begin at the start of the preparation period. In fact, hypertrophy and general power exercises can enhance sports performance, but optimal transfer from football-specific activities also requires football-specific exercise programs [29] in which the biomechanical and neuro-coordinative patterns of sport-specific motor tasks are taxed.

In summary, the analyzed literature suggests that the training of neuromuscular function and its combination with soccer-specific endurance results in improvements in non-specific (e.g., anthropometric characteristics, relative strength, and VO_2_ max) and soccer-specific endurance and neuromuscular parameters (e.g., YYIER, RSA, and sprint).

## Additional file

Additional file 1: Figure S1. The gains in strength and sprint performance of high-level players after 5 to 10 weeks. Squares represent the 10-m distance [2,22,37,38]; circles represent the 20-m distance [37]; rhombi represent the 40-m distance [1,2,6]; + symbols represent the average of all distances; triangles represent the average of the 10-m distance; and lines represent the average of the 40-m distance. **Figure S2.** The gains in strength and jump performance of high-level players after 6 to 10 weeks. Squares represent the squat jump performance (SJ) [1,6,14,22]; triangles represent the countermovement jump (CMJ) performance [2,22,37]; rhombi represent the four bounce test (4BT) performance [6]; lines represent the five jump test [14]; circles represent the average CMJ; x symbols represent the average SJ performance; and + symbols represent the average 4BT performance. **Figure S3.** The gains in strength and change of direction ability of high-level players after 5 to 6 weeks. Squares represent the *t*-test performance [2,38]; circles represent the Zig-Zag test performance [2]; and rhombi represent the Illinois agility test performance [2]. Red-filled triangles represent average of all tests. **Figure S4.** The gains in strength and overall sprint performance of high-level players following traditional resistance exercise programs (TRE; 6 to 10 weeks) and combined programs (COM; 5 to 7 weeks). Filled circles represent the TRE results; empty circles represent the COM results; red-filled circles represent the average TRE [1,2,37]; empty red circles represent the average COM [6,22,38]. **Figure S5.** The gains in strength and overall jump ability of high-level players following traditional resistance exercise programs (TRE; 6 to 10 weeks) and combined programs (COM; 6 to 7 weeks). Blue-filled and unfilled triangles represent the countermovement jump (CMJ) results after TRE and COM, respectively; red-filled and unfilled triangles represent the squat jump (SJ) results after TRE and COM, respectively; green-filled and unfilled triangles represent the four bounce test (4BT) results after TRE and COM, respectively; yellow-filled triangles represent the five jump test (5JT) results after TRE; blue-filled and unfilled circles represent the average CMJ results after TRE [2,37] and COM [22], respectively; red-filled and unfilled circles represent the average SJ results after TRE [1,14] and COM [6,22], respectively; black-filled and unfilled circles represent the average overall jump ability increases after TRE [1,2,6,14,37] and COM [6,22], respectively.
