# Supplementary material for: Strength training in soccer with a specific focus on highly trained players
Source: Sports Med Open. 2015 Apr 2;1:17. doi: 10.1186/s40798-015-0006-z (PMC5005570; doi:10.1186/s40798-015-0006-z)
Supplement: Additional file 1: Figure S1. — The gains in strength and sprint performance of high-level players after 5 to 10 weeks. Squares represent the 10-m distance [2,22,37,38]; circles represent the 20-m distance [37]; rhombi represent the 40-m distance [1,2,6]; + symbols represent the average of all distances; triangles represent the average of the 10-m distance; and lines represent the average of the 40-m distance. Figure S2. The gains in strength and jump performance of high-level players after 6 to 10 weeks. Squares represent the squat jump performance (SJ) [1,6,14,22]; triangles represent the countermovement jump (CMJ) performance [2,22,37]; rhombi represent the four bounce test (4BT) performance [6]; lines represent the five jump test [14]; circles represent the average CMJ; x symbols represent the average SJ performance; and + symbols represent the average 4BT performance. Figure S3. The gains in strength and change of direction ability of high-level players after 5 to 6 weeks. Squares represent the t-test performance [2,38]; circles represent the Zig-Zag test performance [2]; and rhombi represent the Illinois agility test performance [2]. Red-filled triangles represent average of all tests. Figure S4. The gains in strength and overall sprint performance of high-level players following traditional resistance exercise programs (TRE; 6 to 10 weeks) and combined programs (COM; 5 to 7 weeks). Filled circles represent the TRE results; empty circles represent the COM results; red-filled circles represent the average TRE [1,2,37]; empty red circles represent the average COM [6,22,38]. Figure S5. The gains in strength and overall jump ability of high-level players following traditional resistance exercise programs (TRE; 6 to 10 weeks) and combined programs (COM; 6 to 7 weeks). Blue-filled and unfilled triangles represent the countermovement jump (CMJ) results after TRE and COM, respectively; red-filled and unfilled triangles represent the squat jump (SJ) results after TRE and COM, respectively; green-fill [file 40798_2015_6_MOESM1_ESM.pdf]

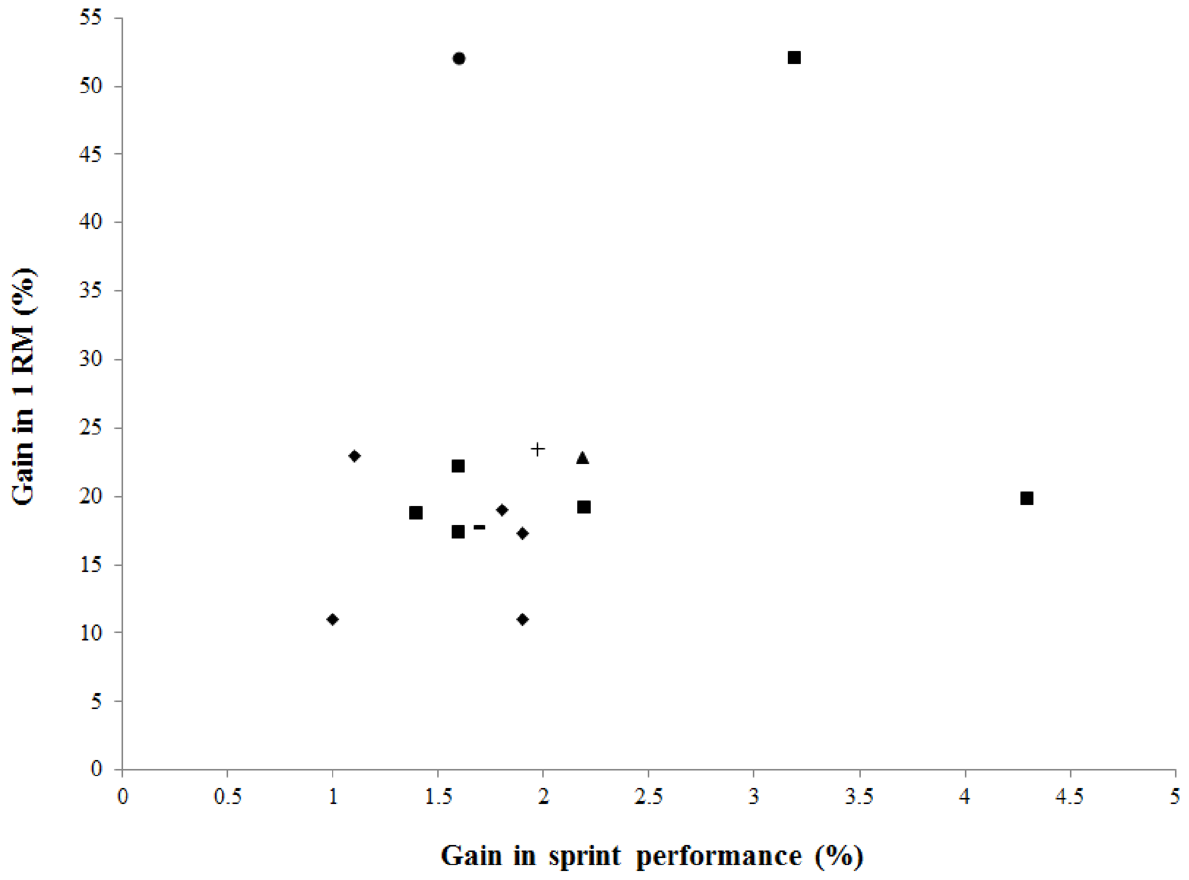

Figure S1 – The gains in strength and sprint performance of high-level players after 5-10 weeks. Squares represent the 10-m distance [2, 22, 37, 38]; circles represent the 20-m distance [37]; rhombi represent the 40-m distance [1, 2, 6]; + symbols represent the average of all distances; triangles represent the average of the 10-m distance; and lines represent the average of the 40-m distance.

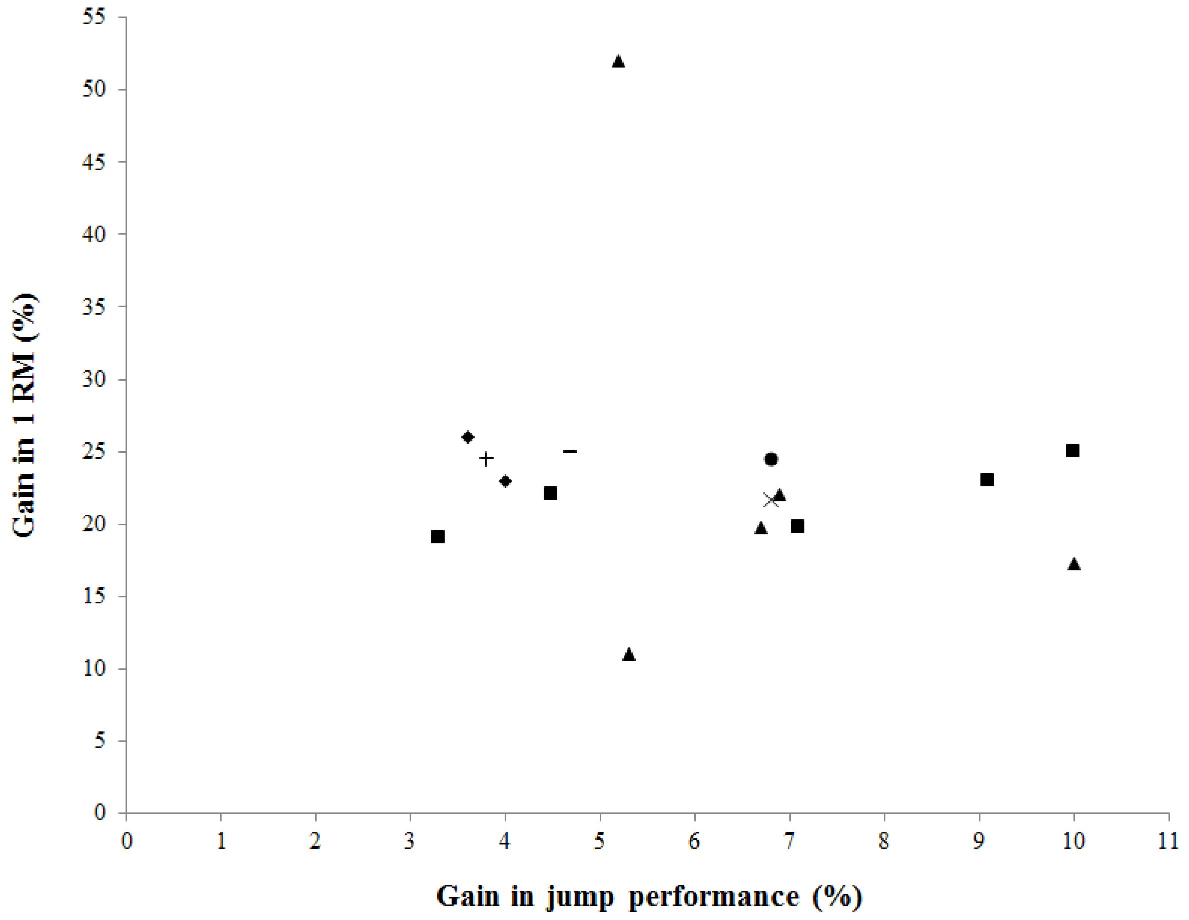

Figure S2 – The gains in strength and jump performance of high-level players after 6-10 weeks. Squares represent the squat jump performance (SJ) [1, 6, 14, 22]; triangles represent the countermovement jump (CMJ) performance [2, 22, 37]; rhombi represent the four bounce test (4BT) performance [6]; lines represent the 5 jump test [14]; circles represent the average CMJ; x symbols represent the average SJ performance; and + symbols represent the average 4BT performance.

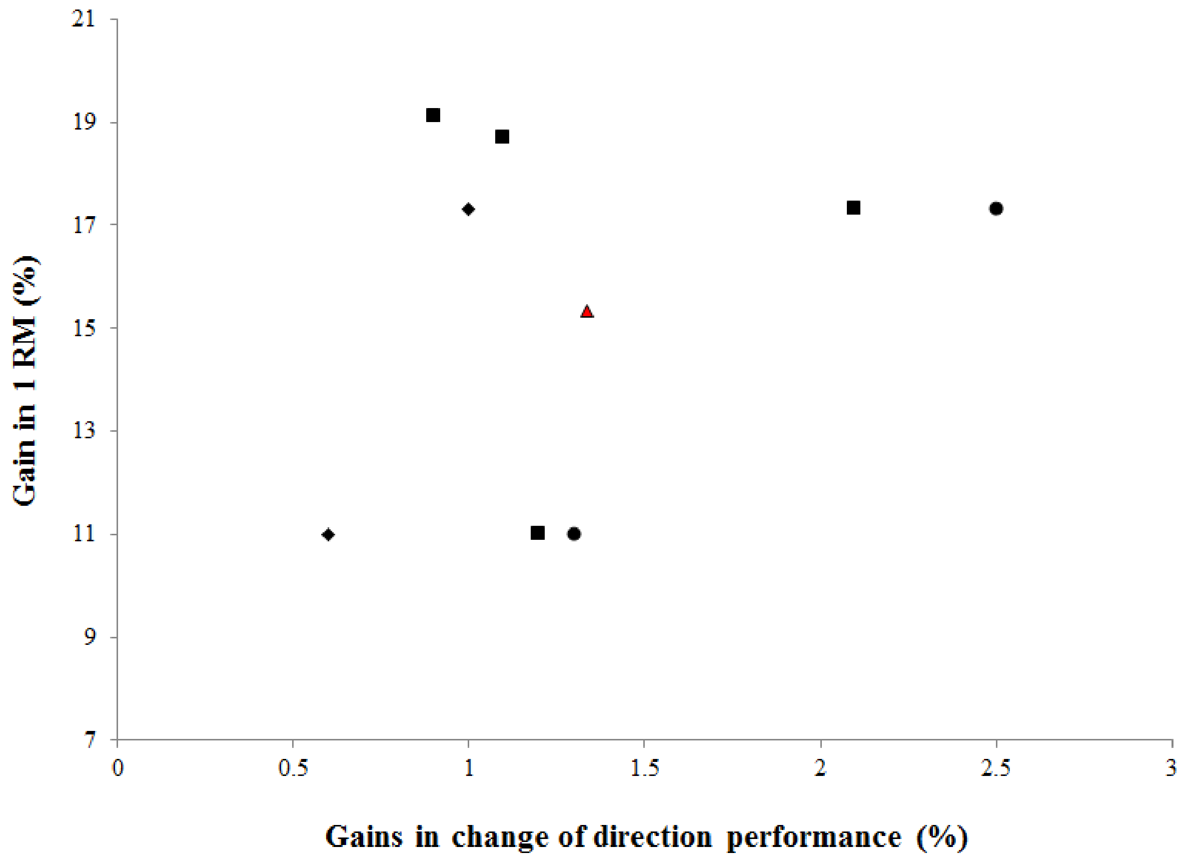

Figure S3 – The gains in strength and change of direction ability of high-level players after 5-6 weeks. Squares represent the T-test performance [2, 38]; circles represent the Zig-Zag test performance [2]; and rhombi represent the Illinois agility test performance [2] red filled triangles represent average of all tests.

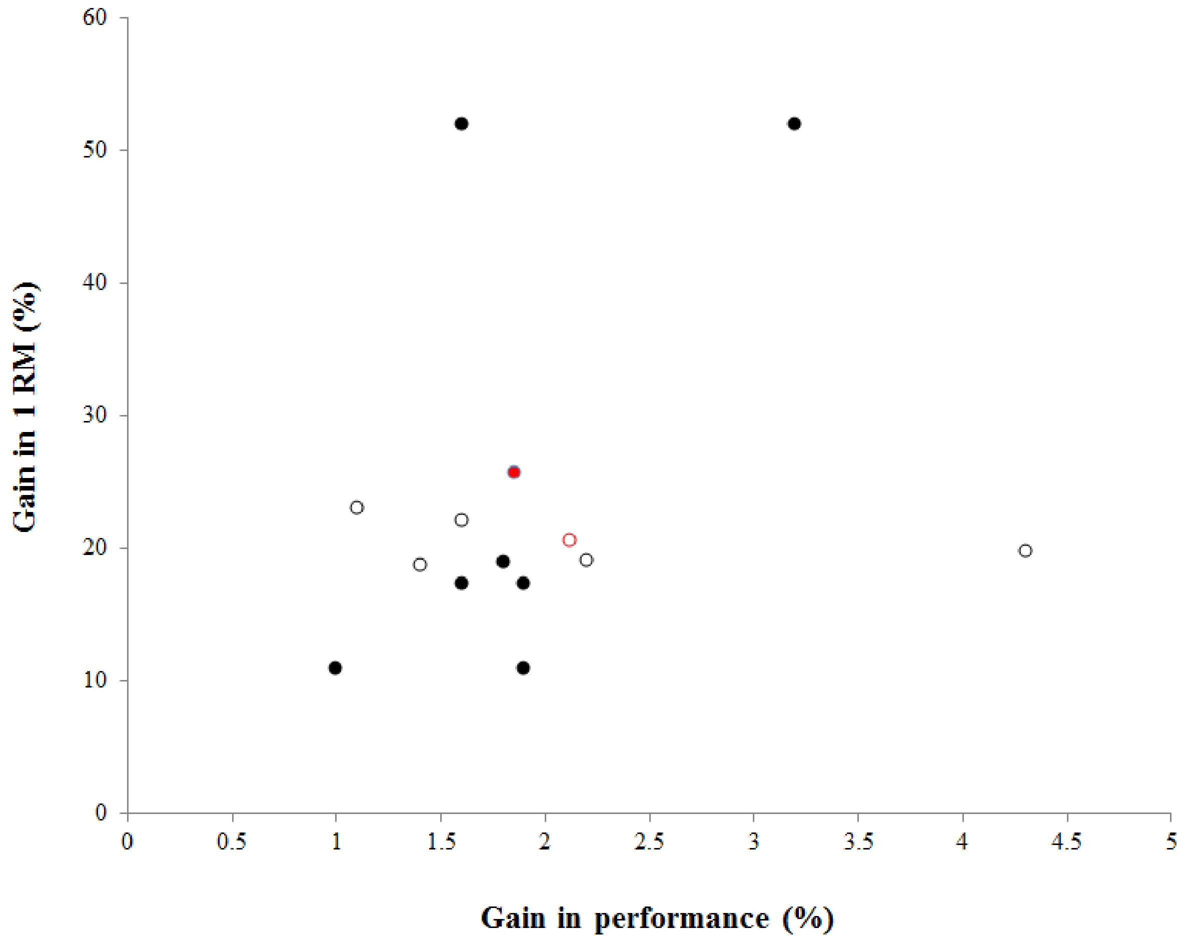

Figure S4 – The gains in strength and overall sprint performance of high-level players following traditional resistance exercise programs (TRE; 6-10 weeks) and combined programs (COM; 5-7 weeks). Filled circles represent the TRE results; empty circles represent the COM results; red filled circles represent the average TRE [1, 2, 37]; empty red circles represent the average COM [6, 22, 38].

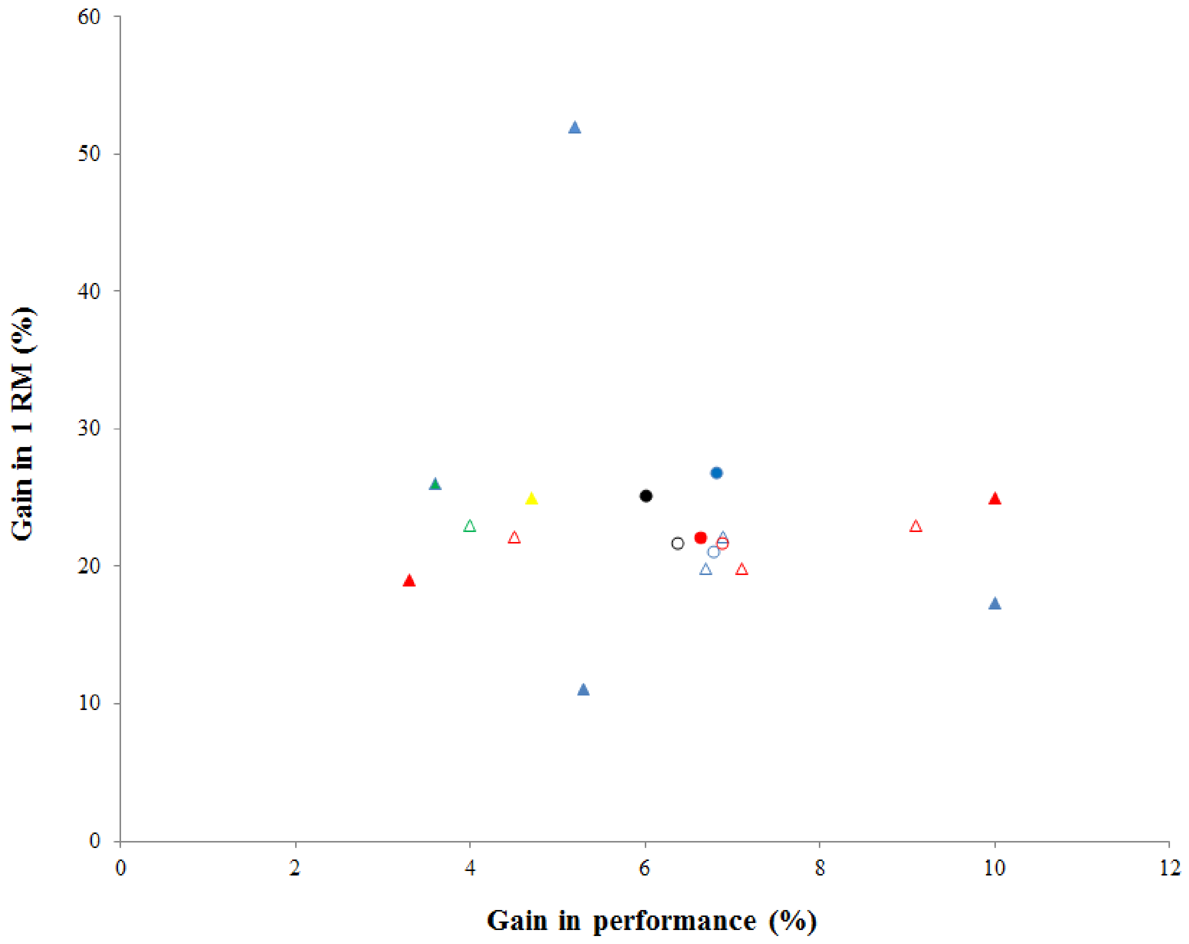

Figure S5 - The gains in strength and overall jump ability of high-level players following traditional resistance exercise programs (TRE; 6-10 weeks) and combined programs (COM; 6-7 weeks). Blue filled and unfilled triangles represent the countermovement jump (CMJ) results after TRE and COM, respectively; red filled and unfilled triangles represent the squat jump (SJ) results after TRE and COM, respectively; green filled and unfilled triangles represent the four bounce test (4BT) results after TRE and COM, respectively; yellow filled triangles represent the five jump test (5JT) results after TRE; blue filled and unfilled circles represent the average CMJ results after TRE [2, 37] and COM [22], respectively; red filled and unfilled circles represent the average SJ results after TRE [1, 14] and COM [6, 22], respectively; black filled and unfilled circles represent the average overall jump ability increases after TRE [1, 2, 6, 14, 37] and COM [6, 22], respectively.
